# Photocatalytic Hydrogen Evolution From Self‐Assembled Stacks of Pd–TCPP and Pt–TCPP

**DOI:** 10.1002/smll.74115

**Published:** 2026-06-18

**Authors:** Jihyeon Kim, Lukas Zdrazil, Xin Zhou, Patrik Schmuki

**Affiliations:** ^1^ Department of Materials Science WW4‐LKO Friedrich‐Alexander‐University of Erlangen‐Nuremberg Erlangen Germany; ^2^ Nanotechnology Centre Centre For Energy and Environmental Technologies VSB – Technical University of Ostrava Ostrava‐Poruba Czech Republic; ^3^ Regional Centre of Advanced Technologies and Materials Olomouc Czech Republic

## Abstract

The present work demonstrates that surfactant‐free precipitation of Pd– and Pt–TCPP in MeOH/H_2_O yields well‐defined supramolecular architectures (µm‐long nanorods) whose photocatalytic function tracks their metal‐dependent photophysics and stacking. Among all tested M–TCPPs (M = Pd, Pt, Zn, Au, and Co), only Pd–TCPP and Pt–TCPP assemble to nanorods that are intrinsically active for H_2_ evolution without any Pt cocatalyst, with Pd–TCPP outperforming Pt–TCPP robustly across the optimal aggregation window (pH ≈ 4–5). In fact, the cocatalyst‐free Pd–TCPP nanorods surpass many other state‐of‐the‐art porphyrin platforms, including Zn–TCPP assemblies operated with high‐loaded Pt cocatalysts, as well as porphyrinic metal–organic frameworks (MOFs). The self‐assemblies display J‐type π–π stacking stabilized by carboxylate hydrogen bonding, producing broadened, red‐shifted light absorption and efficient charge transport. Correlating XRD analysis, spectroscopy, and electrochemistry reveals that compared to Pt–TCPP, Pd–TCPP nanorods exhibit tighter π–π stacking, extended triplet‐state lifetime, and lower charge‐transfer resistance, consistent with more efficient charge separation and faster interfacial electron transfer. Importantly, the approach taken in this work allows for one‐pot, surfactant‐free, co‐catalyst‐free operation of Pd–TCPP nanorods that not only is of low synthetic complexity but also avoids surfactant residues while delivering a high and stable photocatalytic H_2_ production activity under visible illumination.

## 1 Introduction

Porphyrins and their metal complexes are attractive light‐harvesting units and catalysts due to their intense visible absorption (Soret band ∼400–450 nm and Q‐bands 500–650 nm) and favorable excited‐state redox properties [1, 2, 3]. Over the past decade, diverse porphyrin‐based photocatalyst architectures have been explored – from discrete molecular sensitizers [4, 5] to supramolecular assemblies [6, 7], conjugated porphyrin polymers [8], and hybrid composites [9, 10, 11], with a particular focus on uses in photocatalytic H_2_ evolution. In this field, except for attaching porphyrins as antennas or light harvesters on classic semiconductors or electron‐collecting structures [5, 12], porphyrins can be active units in metal–organic frameworks (MOFs) [13, 14], and many porphyrins can be solution‐assembled into unique nanometer‐ to micrometer‐sized structures such as nanospheres [15], nanorods [16], and nanowires [17, 18].

In particular, supramolecular self‐assembly of porphyrin‐derivatives in aqueous media has attracted attention as a simple strategy to organize porphyrins into ordered, directly photocatalytically active architectures. By non‐covalent interactions such as hydrogen bonding, π–π stacking, hydrophilic/hydrophobic interactions, and electrostatic interactions [19], porphyrins readily form aggregates – i.e., various solvent‐antisolvent approaches can be used for synthesis. Zn–TCPP, for example, spontaneously forms aggregates in mixed solvents [20], but the resulting structures are often irregular and poorly crystalline, in part because Zn(II) tends to coordinate axial ligands that disrupt face‐to‐face stacking [21, 22, 23]. Therefore, the self‐assembly of porphyrins into ordered nanostructures often involves surfactants or templates. Gautam et al. demonstrated that CTAB surfactant directs Zn–porphyrin to form nanocubes, nanorods, and microrods in solution [24]. Liu et al. reported that different concentrations of CTAB allow for controlling the size and morphologies of In‐TPP self‐assembly [25]. Such surfactant‐assisted methods have been widely used to produce well‐defined nanoarchitectures.

However, surfactant chemistry not only involves additional synthesis and cleaning steps—thus preventing a true direct self‐assembly of a photocatalyst—but also surfactant residues can interfere with charge transport [26]. More recently, surfactant‐free assemblies of metalloporphyrins have been reported, exploring the possibility of obtaining 1D or 2D ordered nanostructures purely through π–π interactions and hydrogen bonding [20, 27].

Nevertheless, most of these systems still suffer from the drawback that platinum or other co‐catalyst nanoparticles (NPs) must be decorated onto the structure to efficiently catalyze photocatalytic H_2_ generation. In other words, the M‐porphyrin structure acts as light absorber and the added NPs as charge‐transfer and HER‐active centers. For example, Zn–porphyrin nanosphere assemblies without Pt cocatalyst provide only H_2_ evolution efficiency of 0.07 mmol g^−1^ h^−1^, whereas loaded with 3 wt.% Pt cocatalyst, the structure achieved H_2_ evolution rates of 1.2 mmol g^−1^ h^−1^ [20]. Overall, a fully self‐assembled porphyrin photocatalyst that operates neither with any surfactant (or other structure guiding aids) nor with the use of Pt cocatalyst remains highly desirable.

Here, we describe that various 5,10,15,20‐Tetrakis‐(4‐carboxyphenyl)‐21,23H‐porphine (TCPP) and M–TCPP compounds (M = Pd, Pt, Zn, Au, and Co) self‐assemble into various structures but Pd–TCPP and Pt–TCPP form well‐defined 1D µm‐long nanorods in aqueous solution without any surfactant or templating agent. Notably, these Pd–TCPP and Pt–TCPP nanorods are active for H_2_ evolution under visible light without requiring a Pt cocatalyst. Moreover, directly formed from a methanol/water mixture (and the addition of ascorbic acid as sacrificial electron donor), under 450 nm LED irradiation (75 mW cm^−2^), the Pd–TCPP rods achieve a H_2_ production rate of up to 18.5 mmol g^−1^ h^−1^ – a value that is amongst the highest rates reported for porphyrins under comparable illumination conditions and significantly higher than that of analogous Pt–TCPP assemblies (up to 6.6 mmol g^−1^ h^−1^) and far exceeds the activities of Zn–TCPP systems even with Pt cocatalysts (1.3 mmol g^−1^ h^−1^) or Pd–TCPP based 2D‐MOFs (4.4 mmol g^−1^ h^−1^) [13].

## 2 Results and Discussion

**FIGURE 1:**
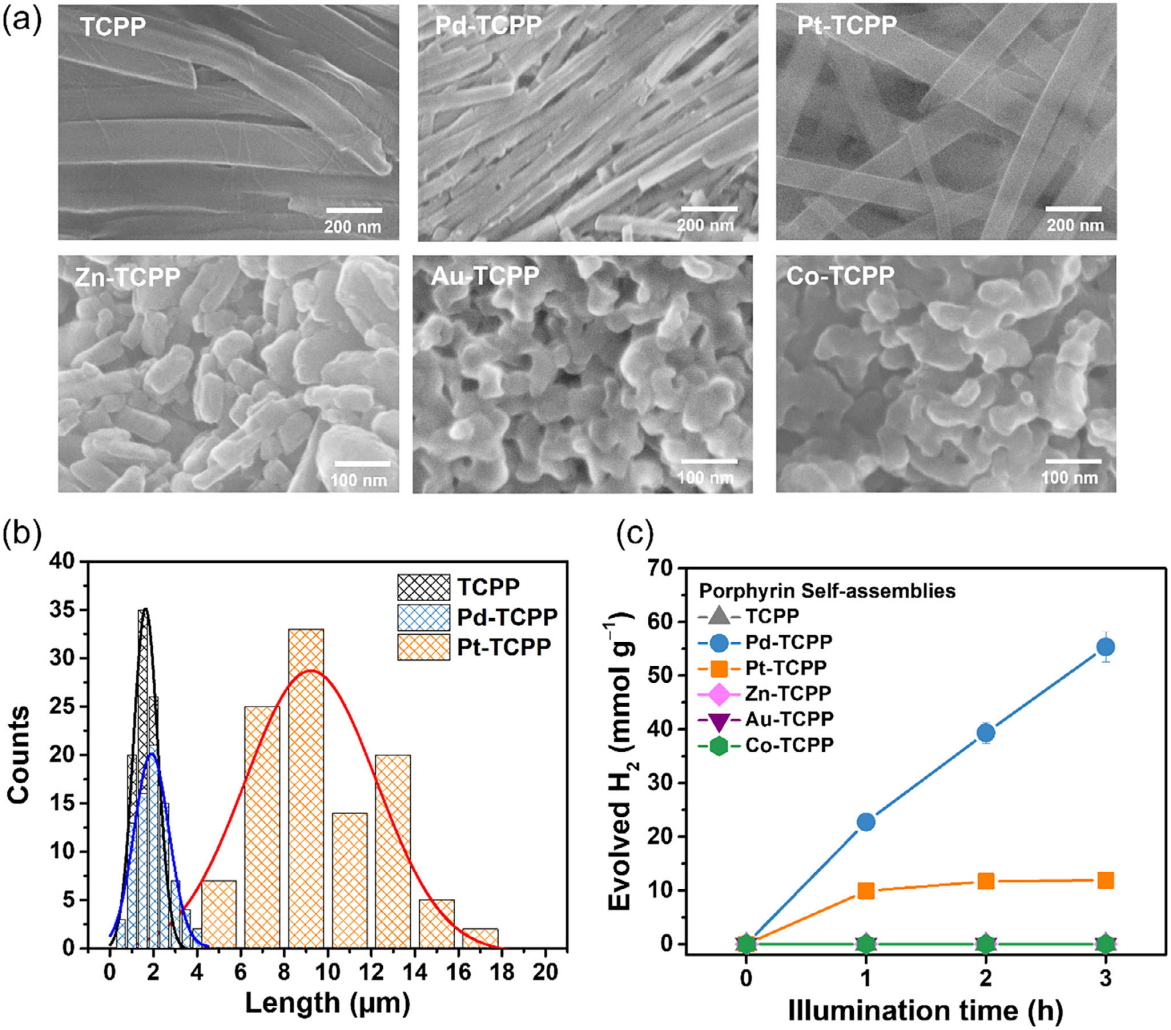
(a) SEM images of TCPP, Pd–TCPP, Pt–TCPP, Zn–TCPP, Au–TCPP, and Co–TCPP stacks. (b) Size distribution of TCPP, Pd–TCPP and Pt–TCPP stacks. (c) Photocatalytic H_2_ evolution (450 nm LED, 75 mW cm^−2^).

In our work, self‐assembled porphyrin structures were prepared by the precipitation approach described in the Experimental section. In short, commercial metal‐free TCPP and metallated TCPPs (M–TCPP, M = Pd, Pt, Zn, Au, and Co) were first dissolved in methanol, followed by the addition of aqueous phase (in which they are insoluble)—the porphyrins then precipitate into distinct microscopic and nanoscopic morphologies, such as shown in Figure 1a.

The scanning electron microscopy (SEM) images in Figure 1a reveal that metal‐free TCPP, Pd–TCPP, and Pt–TCPP form uniform nanorod morphologies. Statistical analysis (Figure 1b) gives typical dimensions of 1.5 ± 0.5 µm in length and 137 ± 6.1 nm in diameter for TCPP; 1.9 ± 1.1 µm in length (with “tails” up to ∼4 µm) and 45.5 ± 1.9 nm in diameter for Pd–TCPP; and 8.8 ± 2.8 µm in length (with tails up to ∼16 µm) and 139.1 ± 7.3 nm in diameter for Pt–TCPP. By contrast, Zn–TCPP, Co–TCPP, and Au–TCPP precipitate as irregular aggregates with characteristic diameters of 40.8 ± 13, 42.6 ± 7, and 60.0 ± 24 nm, respectively. These morphology differences—the wire‐like assemblies of Pd– and Pt–TCPP—can be attributed to metal‐dependent structure‐directing properties [28] and specific intermolecular interactions that govern stacking and lateral cohesion. The formation of porphyrin self‐assembly – particularly in the absence of a surfactant – is affected by various factors, including the porphyrin functional groups, metal ion (e.g., size or in‐/out‐of‐plane coordination behavior), solvent, and pH value. In particular, the metal center can significantly affect the molecular planarity, thus the electronic structure, axial coordination tendency, and intermolecular interactions of the porphyrin unit. These factors strongly influence the self‐assembly pathway and resulting morphology (see discussion in [28, 29, 30, 31]).

These self‐assemblies (stacks) were then evaluated as photocatalysts for H_2_ evolution in the presence of a sacrificial electron donor (0.1 m ascorbic acid). Figure 1c shows the cumulative H_2_ produced under 450 nm LED illumination (75 mW cm^−2^). Among different porphyrin structures, only Pd–TCPP and Pt–TCPP nanorods display measurable photocatalytic H_2_ evolution, reaching 18.5 and 4.0 mmol g^−1^ h^−1^, respectively. In contrast, TCPP, Zn–TCPP, Co–TCPP, and Au–TCPP assemblies yield no detectable H_2_ under identical conditions. Zn–TCPP assemblies only produce H_2_ in the presence of (a high loading of) Pt cocatalyst; this is with an efficiency of 1.3 mmol g^−1^ h^−1^, which is still far less than co‐catalyst‐free Pd–TCPP stacks (Figure S1). In other words, both Pd–TCPP and Pt–TCPP stacks are highly active without deposition of an external cocatalyst.

Notably, Pd–TCPP stacks exhibit substantially higher activity than Pt–TCPP stacks, which contrasts with the conventional expectations for H_2_ evolution catalysts (HER volcano trends)—where Pt outperforms Pd [32]. In order to exclude the possibility that differences in size and aspect ratio determine the activity, we aged Pd–TCPP assemblies in the dark for 2 days (“Pd–TCPP stack 2d”) to increase their dimensions toward those of the Pt–TCPP stacks (Figure S2a). Evidently, the increase in diameter and length upon aging without significant structural or chemical changes (Figure S2b,c), the Pd–TCPP stack 2d sample displays a similar—or even slightly enhanced—HER rate compared to the as‐prepared, slimmer Pd–TCPP rods (Figure S2d), indicating that the superior activity of Pd–TCPP over Pt–TCPP is not a trivial consequence of smaller size.

**FIGURE 2:**
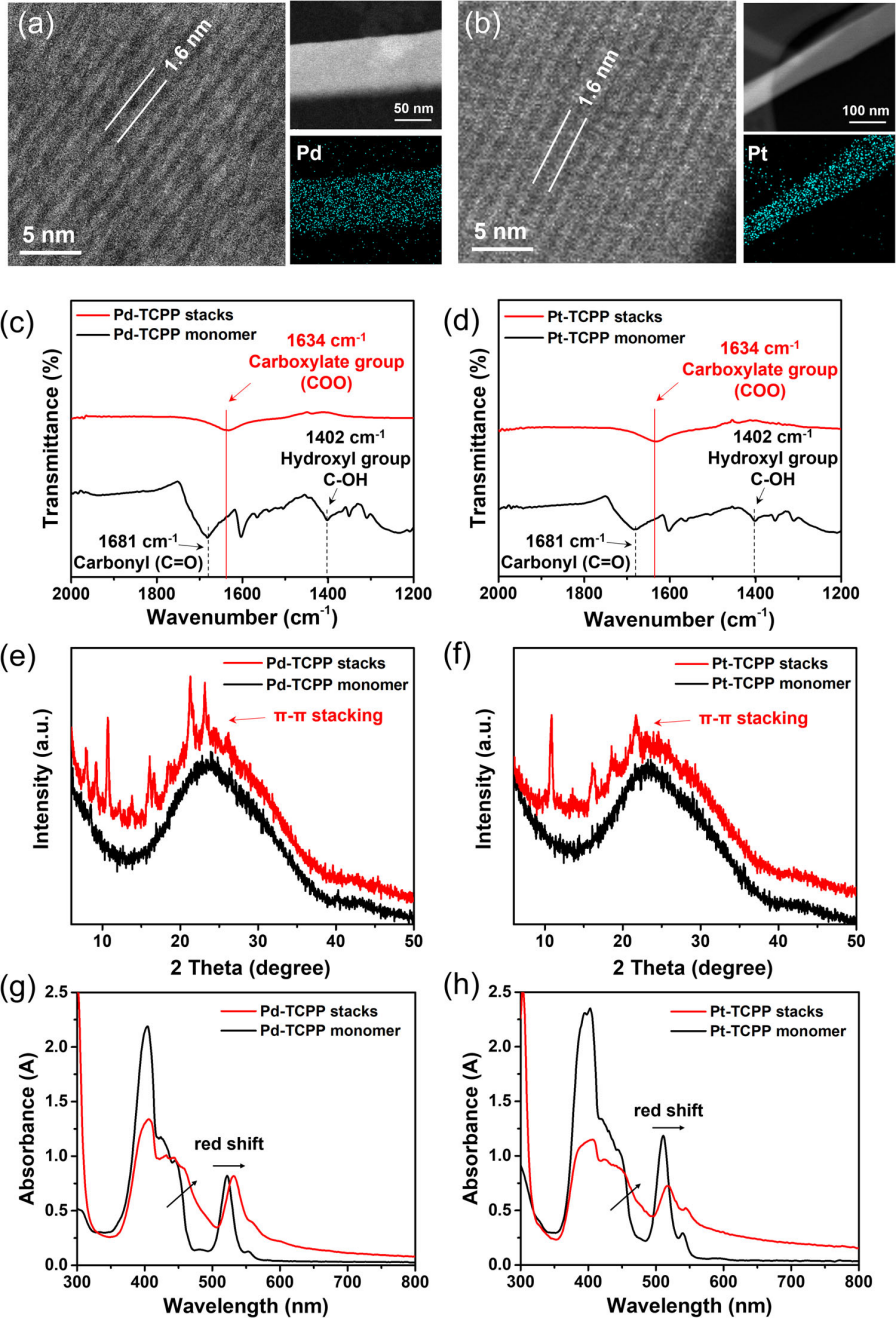
(a,b) HAADF–STEM and STEM–EDX mapping images of Pd–TCPP and Pt–TCPP stacks. (c,d) FT–IR spectra, (e,f) XRD patterns, and (g,h) UV–vis absorption spectra of Pd–TCPP monomer, Pd–TCPP stacks, Pt–TCPP monomer, and Pt–TCPP stacks.

**SCHEME 1:**
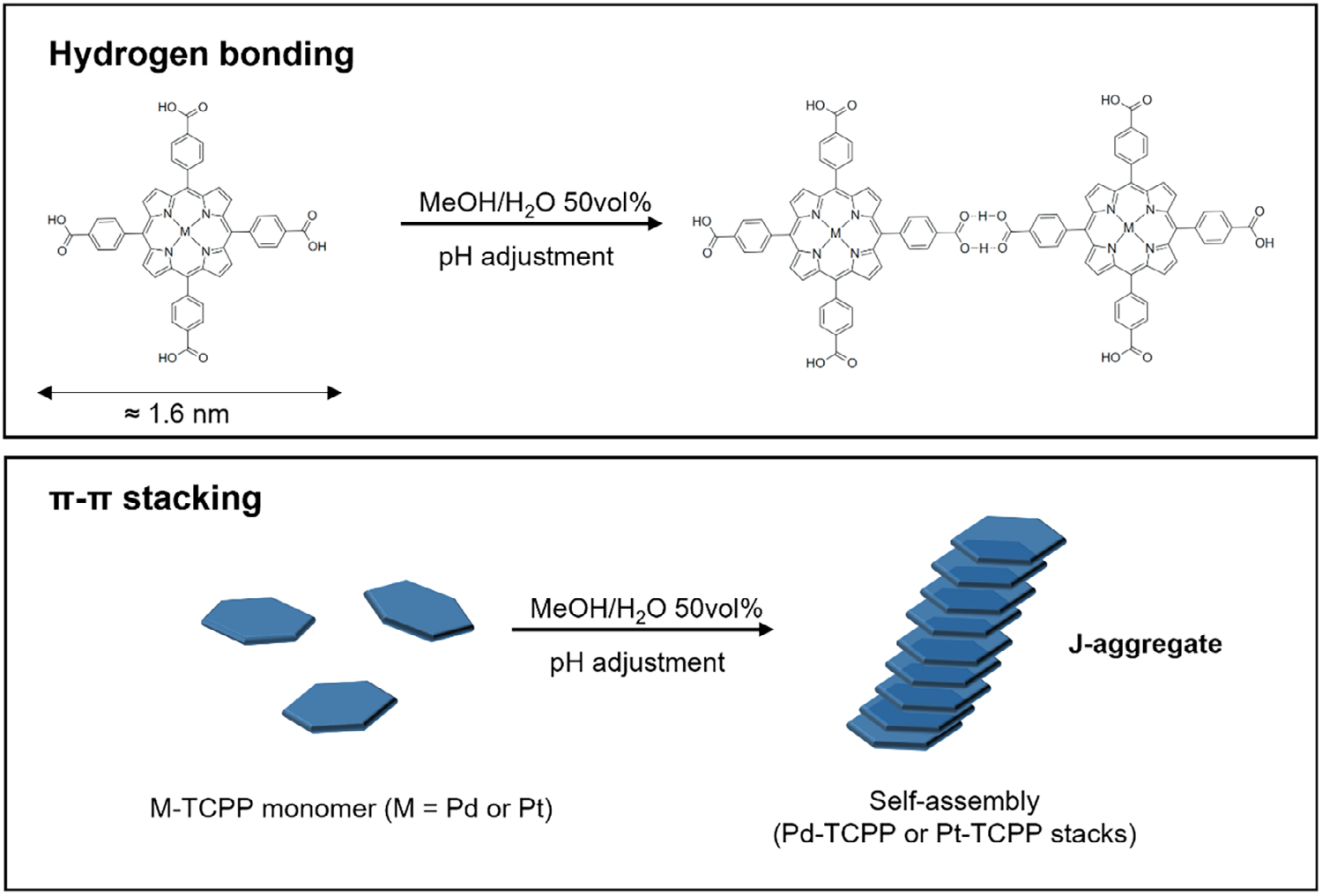
Conceptual illustration of the synthesis of porphyrin self‐assemblies via hydrogen bonding and π‐π stacking.

To elucidate the self‐assembly and stacking structure, we performed high‐angle annular dark‐field scanning transmission electron microscopy (HAADF‐STEM), energy dispersive X‐ray spectroscopy (EDS), X‐ray photoelectron spectroscopy (XPS), Fourier transform infrared (FTIR) spectroscopy, X‐ray Diffraction (XRD), and UV–vis absorption spectroscopy. HAADF‐STEM images (Figure 2a,b) show that both Pd–TCPP and Pt–TCPP nanorods exhibit an average real‐space periodicity of ≈ 1.6 nm, consistent with the molecular dimensions of TCPP, indicating that individual porphyrin units are laterally connected through hydrogen bonding interactions between carboxylic groups as previously reported [33] and depicted in Scheme 1 [34]. Corresponding STEM‐EDS elemental maps (Figure 2a,b; Figure S3) confirm uniform spatial distribution of all elements (i.e., no pop‐out features or metal NP agglomeration). High‐resolution XPS (Figure S4) shows that Pd–TCPP stacks display Pd 3d_5/2_ and 3d_3/2_ peaks at 338.4 and 343.6 eV, respectively, consistent with Pd^2+^ bound in a porphyrin macrocycle [35]. Pt–TCPP stacks display Pt 4f_7/2_ and 4f_5/2_ at 72.7 and 76.1 eV, respectively, in line with Pt^2+^ coordination within a porphyrin [36]. These data also indicate that Pd and Pt remain incorporated in the porphyrin rings without leaching or forming metallic clusters during assembly. Evidence for strong inter‐porphyrin interactions in the assembled structures was further examined by FTIR spectroscopy (Figure 2c,d). Pd–TCPP and Pt–TCPP monomers exhibit bands at 1681 and 1402 cm^−1^, assigned to protonated carboxylic acid groups. Upon assembly into nanowires, these features disappear and a dominant band at 1634 cm^−1^ emerges, accompanied by a weaker symmetric COO^−^ vibration near 1400 cm^−1^, indicating the conversion of free COOH groups into strongly interacting carboxyl/carboxylate environments in the assembled state. This behavior indicates deprotonation and formation of strongly interacting carboxyl/carboxylate motifs within the aggregates. The separation between asymmetric and symmetric COO^−^ stretches is consistent with hydrogen‐bonded or ion‐paired carboxylate networks rather than direct metal–carboxylate coordination, supporting a self‐assembly mechanism stabilized by intermolecular carboxyl interactions [37, 38].

**FIGURE 3:**
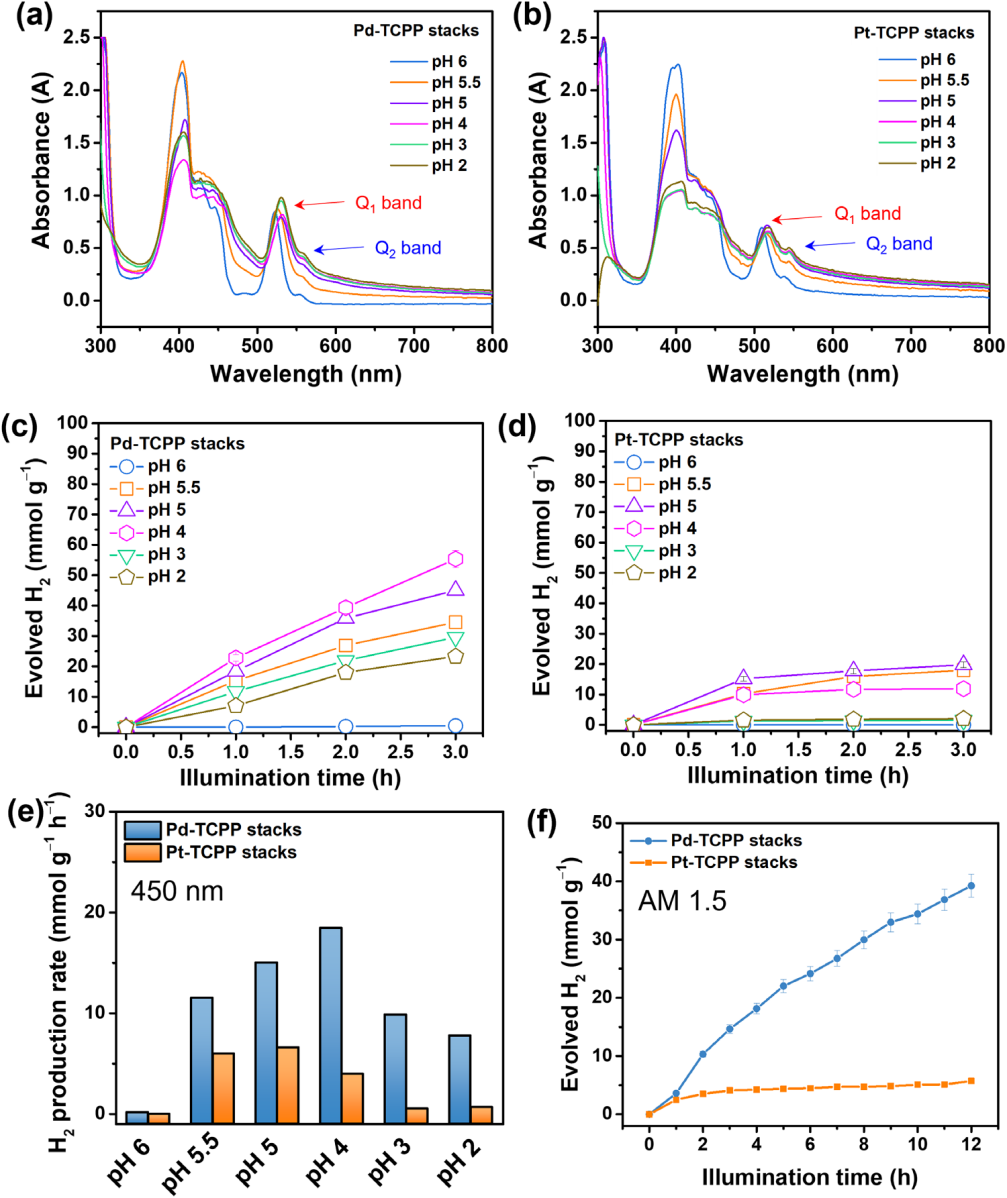
(a,b) UV–vis absorption spectra, (c,d) photocatalytic H_2_ evolution (450 nm LED, 75 mW cm^−2^), and (e) corresponding photocatalytic H_2_ production rate of Pd–TCPP and Pt–TCPP stacks prepared in different pH solutions. (f) Photocatalytic stability test for 12 h under simulated solar light (AM 1.5G, 100 mW cm^−2^) with a 420 nm UV filter of Pd–TCPP and Pt–TCPP stacks prepared in a pH 4 solution.

XRD patterns of Pd–TCPP and Pt–TCPP were recorded before and after agglomeration (Figure 2e,f). As expected, the Pd–TCPP and Pt–TCPP monomers exhibit no clear diffraction peaks. Both Pd–TCPP and Pt–TCPP stacks reveal broad but distinct reflections at 2θ ≈ 5.6°, 10.7°, 16.7°, and 26.5°, corresponding to d‐spacings of 1.6, 0.83, 0.53, and 0.34 nm, respectively. The low‐angle feature at 5.6° matches the periodicity observed in HAADF‐STEM (≈1.6 nm) and is attributed to the lamellar repeat distance between porphyrin layers linked via hydrogen bonding between carboxyl groups. The higher‐angle reflection near 26.5° corresponds to the π–π stacking distance typical for slipped J‐type aggregates [39]. Compared to Pt–TCPP stacks, Pd–TCPP stacks show more intense and slightly sharper reflections, indicating a higher degree of molecular ordering and extended coherence length along the stacking axis. These structural characteristics are consistent with the SEM and TEM observations (Figures 1b and 3a,b).

UV–vis absorption spectroscopy further reveals the collective optical response of the assemblies. In MeOH solution, Pd–TCPP monomer exhibits a Soret band at 404 nm and Q bands at 522 and 555 nm, while Pt–TCPP monomer shows a Soret band at 403 nm and Q bands at 511 and 540 nm. After assembly into rods (Figure 1a), both Pd–TCPP and Pt–TCPP stacks display broadened visible absorption and red‐shifted Q bands relative to the monomers (Figure 2g,h). Such red shifts are characteristic of strong dipole–dipole coupling within π‐conjugated arrays and indicate slipped π‐π stacking (typically J‐type aggregation), in which porphyrin units adopt head‐to‐tail packing [40, 41, 42, 43]. Overall, Pd–TCPP and Pt–TCPP stacks share a common microscopic stacking motif–lateral association via (carboxyl/carboxylate) hydrogen‐bond networks coupled with cofacial π–π interactions along the rod axis—while their optical absorption at the photocatalysis wavelength (450 nm) is essentially identical (Figure 2g,h).

As hydrogen bonding is a key driving force for self‐assembly and for extending electronic coupling over porphyrin aggregates, we next examined how the pH influences structure formation and photocatalytic activity. Given that the apparent p*K*_a_ of TCPP carboxylic acids is 4–5 [44, 45], the –COOH groups become increasingly deprotonated (–COO^−^) at pH ≥ 5, which enhances aqueous solubility and suppresses intermolecular aggregation. Consistent with this, in a relatively neutral media (pH 5.5–6), the absorption spectrum of the precipitates resembles those of the monomers (Figures 2g,h and 3a,b), in line with a clear visible onset of the aggregation at more acidic conditions. Indeed, Pd–TCPP precipitates prepared across a pH series confirm efficient aggregation at pH < 5 (Figure S5). As shown in Figure S6, the *λ*_max_ values of both Q bands of Pd–TCPP and Pt–TCPP stacks gradually red‐shift as pH is lowered from 6 to 2. Such behavior is well‐established for slipped, head‐to‐tail excitonic coupling (J‐aggregate) in porphyrin systems [39]. Moreover, as the pH decreases, the absorption features not only red‐shift but also broaden, reflecting increased dipole–dipole coupling and enhanced exciton delocalization within aggregates (pH < p*K*_a_) [46, 47].

Photocatalytic H_2_ evolution as a function of pH (Figure 3c–e; 450 nm LED, 75 mW cm^−2^) shows negligible activity at pH 6 for both Pd–TCPP and Pt–TCPP stacks. Upon lowering the pH, the H_2_ evolution rate increases and maximizes at pH 4–5 (18.5 mmol g^−1^ h^−1^ for Pd–TCPP; 6.6 mmol g^−1^ h^−1^ for Pt–TCPP) but declines again at pH < 4. The loss of activity in strong acidic media is attributed to demetallation/metal leaching from the porphyrin cores. In support, XPS N1s spectra of Pd–TCPP self‐assemblies exposed to pH 2–3 (Figure S7) show two components: a signal at 398.9 eV assigned to metalated porphyrinic N (Pd–N) and a higher‐binding‐energy feature near 402.5 eV consistent with protonated nitrogen species, indicative of loss of Pd from the macrocycle and concomitant protonation under highly acidic conditions (NH formation in XPS) [48]. This demetallation may contribute to the activity loss at pH 2–3 despite sufficient J‐aggregate. We note that a freshly prepared M–TCPP precipitate in the presence of ascorbic acid (0.1 m) exhibits a solution pH of 3–4, which fortuitously lies within the optimal window for photocatalytic H_2_ evolution. For further reference, we also carried out photocatalytic H_2_ evolution of metal‐free TCPP stacks with Pt and Pd cocatalyst (Figures S8 and S9). These stacks do not produce H_2_ efficiently, even if a cocatalyst is present in solution (this is simulating de‐metalation of the porphyrin in solution).

To validate that the above findings on Pd– and Pt–TCPP are excitation‐wavelength independent, we also measured H_2_ evolution under 520 nm irradiation (chosen to directly excite the Q‐band; Figure 3a,b). As shown in Figure S10, both Pd–TCPP and Pt–TCPP stacks display the same pH dependence under 520 nm as under 450 nm: the maximum H_2_ rate occurs for precipitates formed at pH 4–5, and at all pH values, the Pd–TCPP stacks outperform the Pt–TCPP stacks. Finally, we assessed the operational stability of assemblies formed at pH 4 under continuous irradiation for 12 h (AM 1.5G, 100 mW cm^−2^ with a 420 nm cut‐off filter). As summarized in Figure 3f, Pd–TCPP stacks maintain stable activity and deliver a > 9‐fold higher H_2_ evolution than Pt–TCPP stacks over the entire test, whereas Pt–TCPP exhibits a progressive decay in activity. To evaluate the structural stability, we characterized them further after photocatalytic HER. SEM images and XRD patterns (Figure S11a,b) show that the catalyst preserves its nanorod morphologies and crystallinity without any reorganization after reaction. Furthermore, the XPS N 1s and Pd 3d spectra (Figure S11c,d) confirm not only structural stability, but also the retention of Pd atoms within the porphyrin macrocycle through Pd─N coordination, with no evidence of uncoordinated N species (──NH─ or ═N─) or Pd NPs, nor any free Pd ions from solution analysis (see Figure S12) arising from metal leaching. Likewise, as there is no evidence of Pt metal leaching into solution or Pt nanoparticle formations on the stacks, as confirmed by SEM, XPS, and solution analysis (Figure S13), we speculate that the plateau is related to the lower structural order/integrity of the Pt–TCPP stacks Particularly, the weaker high‐angle XRD reflection observed for Pt–TCPP stacks (Figure 2e,f) suggest less tight π‐π stacking and a lower degree of structural order than Pd–TCPP stacks. This may lead to weakening of the in situ ordering and a less efficient charge transfer during prolonged photocatalysis.

**FIGURE 4:**
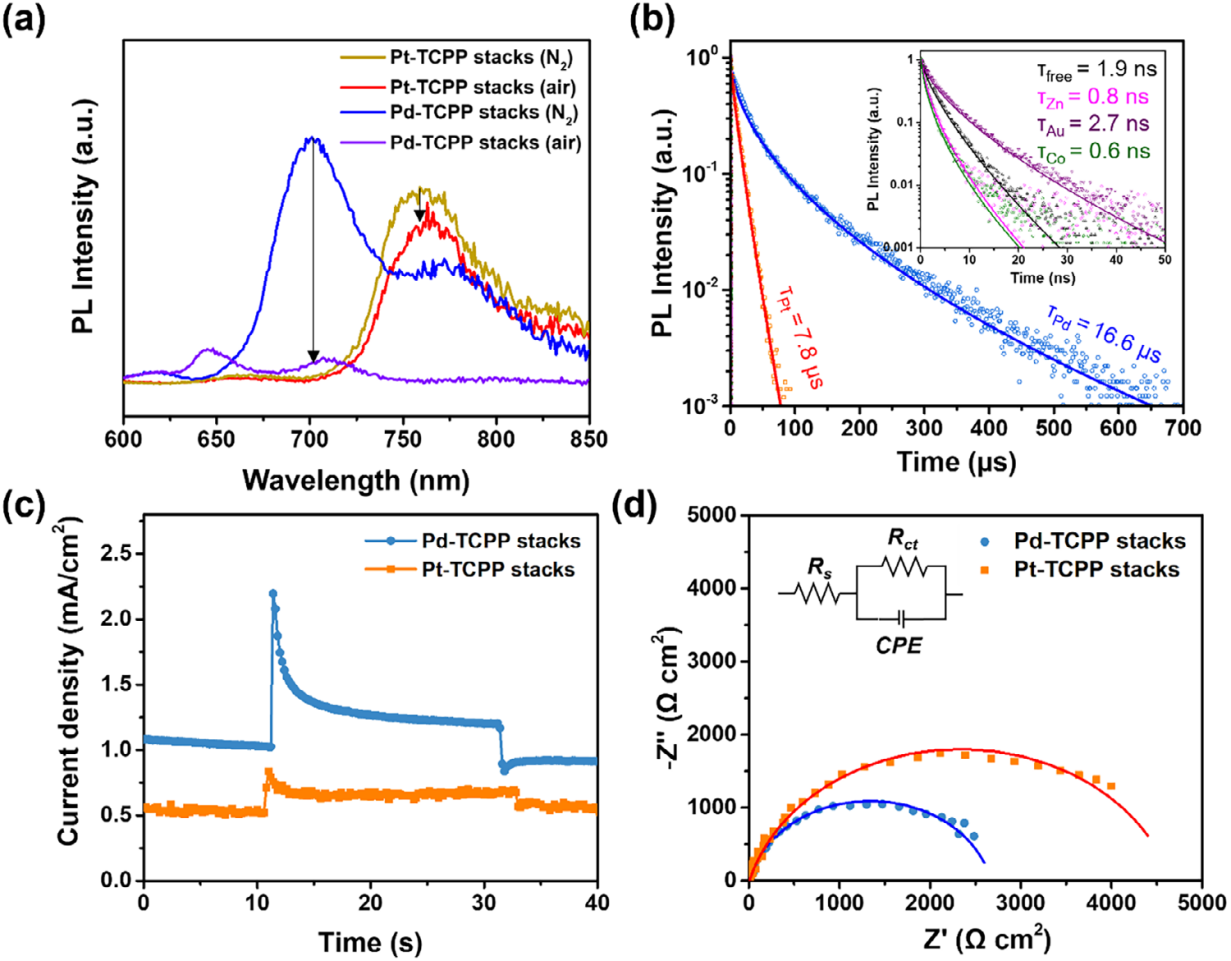
(a) PL emission spectra of Pd–TCPP and Pt–TCPP stacks under N_2_ and air atmosphere. (b) Time‐resolved PL spectra (scatter) and fitted data (solid line) of all the stacks collected at the corresponding PL maxima under N_2_ atmosphere. (c) Photocurrent response of Pd‐TCPP and Pt‐TCPP stacks as a photoelectrode at 0.5 V (vs. Ag/AgCl) in 0.1 m Na_2_SO_4_ aqueous electrolyte under illumination (λ = 450 nm, 500 mW cm^−2^). (d) EIS plots of Pd–TCPP and Pt–TCPP stacks electrode recorded in the frequency range of 10^−1^–10^6^. Solid lines represent software fitting of the data with an equivalent circuit model shown in the top‐left corner.

Across a wide range of experimental conditions—varying hydrogen‐bonding strength (via pH), potential metal leaching in strongly acidic media, and excitation wavelength—Pd–TCPP stacks consistently deliver markedly higher H_2_ evolution rates than Pt–TCPP stacks, and clearly outperform all other self‐assembled M–TCPPs tested. A plausible origin for the high efficiency is the so‐called heavy‐atom effect, where incorporation of Pd(II) or Pt(II) into the porphyrin macrocycle enhances spin–orbit coupling and promotes intersystem crossing (ISC), thereby populating long‐lived triplet states that are beneficial for charge separation and proton reduction [13, 49, 50, 51]. To assess this for our assemblies, we compared photoluminescence (PL) measurements, time‐resolved PL spectra, photocurrent transients, and electrochemical impedance spectroscopy (EIS) for Pd–TCPP, Pt–TCPP, and the other M–TCPP stacks shown in Figure 1. Clearly, the PL spectra (Figure 4a) of Pd–TCPP stacks are strongly quenched under air conditions relative to N_2_, indicating that Pd–TCPP stacks undergo efficient ISC from the singlet to the triplet state. This oxygen‐sensitive emission demonstrates that the excited states of these aggregates are dominated by long‐lived triplets. In contrast, the spectra of Pt–TCPP stacks are only slightly quenched under aerated conditions, indicating inefficient ISC and a limited contribution of triplet states. Even more notably, the other stacks (metal‐free, Zn, Au, and Co) show virtually no quenching under aerated conditions compared to N_2_ (Figure S14), indicating that the emission mainly originates from short‐lived singlet excited states, not from long‐lived triplet states. Likewise, the time‐resolved PL measurements (Figure 4b) further reveal that Pd–TCPP stacks exhibit a 2 times longer lifetime (τ_Pd_ = 16.6 µs) than Pt–TCPP stacks (τ_Pt_ = 7.8 µs), in line with a higher triplet yield expected for Pd porphyrins [51, 52]. Compared to Pd–TCPP and Pt–TCPP stacks (lifetime in the range of ∼ µs), the other samples (Zn, Au, and Co–TCPP stacks) exhibit much shorter lifetimes (in the range of ∼ ns), which is also in line with our experimental data that Pd–TCPP and Pt–TCPP stacks show high H_2_ evolution activity, whereas Zn–, Au–, and Co–TCPP do not produce H_2_. Transient photocurrent measurements (Figure 4c) on photoelectrodes further show that Pd–TCPP stacks generate a larger photocurrent response under visible irradiation, indicating more efficient separation and transport of photogenerated carriers than in Pt–TCPP stacks. Similarly, EIS Nyquist plots (Figure 4d) reveal a smaller semicircle for Pd–TCPP stacks, indicative of lower interfacial charge‐transfer resistance. Fitting to a classical Randles equivalent circuit (Table S1) yields *R*_ct_ = 2661 Ω cm^2^ for Pd–TCPP stacks versus *R*_ct_ = 4645 Ω cm^2^ for Pt–TCPP stacks, corroborating that the Pd assemblies support faster interfacial electron transfer than their Pt counterparts. Therefore, the combined results – the PEC, PL, time‐resolved PL, and EIS data – support that the superior photocatalytic H_2_ evolution activity of Pd–TCPP stacks is associated with more favorable charge behavior in both the bulk assembly and the interfacial region.

All above characterization shows that among differently metalated porphyrin stacks, Pd–TCPP stacks exhibit remarkably higher efficiency in photocatalytic H_2_ evolution despite of a comparable morphology to Pt–TCPP stacks in line with its characteristic photophysical properties and namely a long‐lived triplet state. Moreover, it is noteworthy and of considerable practical significance that Pd–TCPP stacks are obtained by a simple, facile precipitation step and can be used directly as a photocatalytic system. This is, in one pot, one may dissolve the porphyrin, precipitate the stacks, and add ascorbic acid as a sacrificial agent, which places the stacks in a stable aggregate regime and within the optimal activity window for efficient H_2_ production. In other words, the active system is achieved without time‐consuming multistep syntheses and separation, without surfactant or templates, and without noble‐metal cocatalysts such as Pt NPs, in contrast to many prior reports on comparable systems (Table S2).

In comparison with other porphyrin‐based self‐assembled structures or Pd–TCPP/Pt–TCPP‐based 2D MOFs, the cocatalyst‐free Pd–TCPP stacks achieve H_2_ evolution rates (18.8 mmol g^−1^ h^−1^) that surpass representative literature values (4.4 mmol g^−1^ h^−1^) obtained from MOFs constructed from the same Pd–TCPP unit (Figure S9 and Table S2) [13]. This suggests that the ordered stacking provided in the nanorod aggregate presented here provides efficient charge transport and, together with the long‐lived triplet state is the basis for the superior photocatalytic H_2_‐evolution activity observed for Pd–TCPP.

## 3 Conclusion

In summary, we establish a one‐step, surfactant‐free protocol to construct self‐assembled nanostructures of TCPP and M–TCPP (M = Pd, Pt, Zn, Au, Co) directly in MeOH/H_2_O. The resulting aggregates exhibit broadened, red‐shifted absorption relative to their monomers, consistent with hydrogen‐bond‐assisted π–π (J‐type aggregation) stacking. When evaluated for photocatalytic H_2_ evolution without any noble‐metal cocatalyst, only Pd–TCPP and Pt–TCPP nanorods are active, with Pd–TCPP consistently delivering the highest rates under 450 or 520 nm excitation and under simulated solar light (AM 1.5, λ > 420 nm) across the aggregation window (pH ≈ 4–5). Activity declines only at strongly acidic pH, where partial demetallation can occur. Spectroscopic and electrochemical probes (PL quenching, transient photocurrent, EIS) indicate more efficient charge separation and lower interfacial resistance in Pd–TCPP than in Pt–TCPP, aligning with a higher stacking order and the longer‐lived triplet manifold of Pd porphyrins. Taken together, these results identify Pd–TCPP self‐assemblies as a rare, co‐catalyst‐free porphyrin platform that provides facile, one‐pot preparation with a state‐of‐the‐art H_2_‐evolution performance.

We believe that the design rules articulated here—the surfactant‐free synthesis of square‐planar porphyrins that form ordered 1D assemblies—provide a key result for the further development of a wide variety of metal‐center‐tuned photocatalysts.

## Conflicts of Interest

The authors declare no conflicts of interest.

## Supporting information




**Supporting File**: smll74115‐sup‐0001‐SuppMat.docx.

## Data Availability

The data that supports the findings of this study are available in the supplementary material of this article.
