# Supplementary material for: Photocatalytic Hydrogen Evolution From Self‐Assembled Stacks of Pd–TCPP and Pt–TCPP
Source: Small. 2026 Jun 18;22(43):e74115. doi: 10.1002/smll.74115 (PMC13432670; doi:10.1002/smll.74115)
Supplement: Supplementary file 1 — Supporting File: smll74115‐sup‐0001‐SuppMat.docx. [file SMLL-22-e74115-s001.docx]

***Supporting Information***

**Photocatalytic Hydrogen Evolution from Self-assembled Stacks of Pd**–**TCPP and Pt**–**TCPP**

Jihyeon Kim^1^, Lukas Zdrazil^2,3^, Xin Zhou^1^, and Patrik Schmuki^1,3^*

^1^Department of Materials Science WW4-LKO, Friedrich-Alexander-University of Erlangen-Nuremberg, Martensstrasse 7, 91058 Erlangen, Germany

^2^Nanotechnology Centre, Centre for Energy and Environmental Technologies, VSB – Technical University of Ostrava, 17. listopadu 2172/15, 708 00 Ostrava-Poruba, Czech Republic.

^3^Regional Centre of Advanced Technologies and Materials, Šlechtitelů 27, 78371 Olomouc, Czech Republic

^*^Corresponding author details

E-mail: [schmuki@ww.uni-erlangen.de](mailto:schmuki@ww.uni-erlangen.de)

**1. Experimental section**

**1.1 Materials**

5,10,15,20-Tetrakis-(4-carboxyphenyl)-porphine-Pd(II) (Pd–TCPP), 5,10,15,20-Tetrakis-(4-carboxyphenyl)-porphine-Pt(II) (Pt–TCPP), 5,10,15,20-Tetrakis-(4-carboxyphenyl)-porphine-Zn(II) (Zn–TCPP), 5,10,15,20-Tetrakis-(4-carboxyphenyl)-porphine-Co(II) (Co-TCPP), 5,10,15,20-Tetrakis-(4-carboxyphenyl)-porphine-Au(III) chloride (Au–TCPP), and 5,10,15,20-Tetrakis-(4-carboxyphenyl)-21,23H-porphyrin (TCPP) were purchased from Por-Lab GmbH. Palladium (II) sodium chloride (Na_2_PdCl_4_), L-Ascorbic acid, dimethylglyoxime, tin chloride (SnCl_2_) and Nafion were purchased from Sigma-Aldrich. Methanol (MeOH, 99.9%), Ethanol (EtOH, 99.8%), hydrochloric acid (HCl, 37%), sodium hydroxide (NaOH, 98%), and sodium sulfate (Na_2_SO_4_, 99%) were purchased from Carl Roth. Hexachloroplatinic(IV) acid (H_2_PtCl_6_, 40% Pt) was purchased from Metakem. All chemicals were used as received.

**1.2 Synthesis of porphyrin self-assembly**

1 mg of TCPP or M–TCPP (M = Pd, Pt, Zn, Au, Co) was dissolved in 5 mL of methanol and sonicated for 10 min. 5 mL of aqueous solution was added to the dissolved porphyrin solution. The obtained porphyrin self-assemblies were either collected by centrifugation for surface characterization or directly used for photocatalytic H_2_ evolution after addition of a sacrificial agent (0.1 M ascorbic acid).

**1.3 Synthesis of Pd–TCPP stack 2 d**

1 mg of Pd–TCPP was dissolved in 5 mL of methanol and sonicated for 10 min. 5 mL of aqueous solution was added to the dissolved porphyrin solution. The obtained porphyrin self-assembly was purged with Ar gas for 15 min, then the sealed container was kept in the dark for 2 days while stirring.

**1.4 Photocatalytic H_2_ evolution of porphyrin self-assembly**

1 mg of TCPP or M–TCPP (M = Pd, Pt, Zn, Au, Co) was dissolved in 5 mL of methanol and sonicated for 10 min. 5 mL of sacrificial agent aqueous solution (0.1 M ascorbic acid) was added to the dissolved porphyrin solution. For the pH-dependent activity test, the pH was adjusted with NaOH or HCl. For the test with cocatalyst, 3wt% H_2_PtCl_6_ or Na_2_PdCl_4_ was added. After Ar gas purging for 15 min, the quartz tube was sealed and irradiated by a 450 nm LED (75 mW/cm^2^), 520 nm laser (1.2 W/cm^2^), or Xe lamp (100 mW/cm^2^) with a 420 nm UV filter under stirring (1200 rpm). Photocatalytic hydrogen evolution was measured by gas chromatograph (GC, GCMS-QO2010SE, Shimadzu). No H_2_ evolution was detected in the blank experiment conducted without the catalyst, confirming that ascorbic acid does not decompose to generate hydrogen under the reaction conditions. Repeated measurements show an error of less than 5%.

**1.5 Photoelectrochemical and electrochemical performance evaluation**

Photoelectrochemical measurements were performed in a 3-electrode cell comprising porphyrin self-assembly as a working electrode, a Pt sheet as a counter electrode, and an Ag/AgCl reference electrode. Photocurrent transient curves were recorded at 0.5 V (vs. Ag/AgCl) in 0.1 M Na_2_SO_4_ under illumination (λ= 450 nm, 500 mW/cm^2^) using Autolab PG-STAT302N potentiostat/galvanostat. Electrochemical Impedance Spectra (EIS) were recorded in the frequency range of 10^-1^-10^6^ Hz at -0.5 V (vs. Ag/AgCl) in 0.1 M Na_2_SO_4_ using Zahner IM6 (Zahner Elektrik, Kronach, Germany). The obtained EIS plots were fitted using the EIS Spectrum Analyzer software to extract the electrical parameters of the electrodes. For the preparation of Pd–TCPP and Pt–TCPP stack electrodes, 2 mg of self-assembly powder was dissolved in 40 µL of 5wt.% Nafion, 80 µL of H_2_O, and 80 µL of ethanol. After sonication for 10 min, 40 µL of the dispersed solution was applied to a glassy carbon electrode.

**1.6 Pd ion leaching test (DMG colorimetric assay)**

After using Pd-TCPP stacks as a photocatalyst, the reaction electrolyte was collected by centrifugation (9000 rpm, 20 min). The solution was adjusted to an acidic condition (pH 2) with HCl prior to the DMG test. A dimethylglyoxime (DMG) reagent was prepared by dissolving 20 mg DMG in 20 mL of ethanol. For the leaching test, 2 mL of the electrolyte was mixed with 1 mL of the DMG solution. After 10 min, the resulting solution was analyzed by UV-vis spectroscopy. For quantitative analysis, Pd standard solutions were prepared from Na_2_PdCl_4_ (0.05 – 0.4 mM) in 0.5 M HCl and reacted with DMG reagent to generate a calibration curve. Absorbance was recorded at 480 nm, and Pd concentration was determined based on the calibration curve.

**1.7 Pt ion leaching test**

After using Pt-TCPP stacks as a photocatalyst, the reaction electrolyte was collected by centrifugation (9000 rpm, 20 min). SnCl_2_ solution was respectively prepared in HCl (6 M). For the leaching test, 2 mL of the electrolyte was mixed with 1 mL of the SnCl_2_ solution. After 1 h, the resulting solution was analyzed by UV-vis spectroscopy. For quantitative analysis, Pt standard solutions were prepared from H_2_PtCl_6_ (0.05 – 0.4 mM) in 6 M HCl and reacted with SnCl_2_ reagent to generate a calibration curve. Absorbance was recorded at 403 nm, and Pt concentration was determined based on the calibration curve.

**2. Characterization**

The morphology of catalysts was characterized by field-emission scanning electron microscopy (SEM, S-4800, Hitachi, Japan) and high-resolution transmission electron microscopy (HR-TEM, CM 30 TEM/STEM, Philips). Fourier-transform infrared spectroscopy was used to identify the presence of hydrogen bonding of porphyrin self-assembly (FTIR, Jasco FT/IR-4700). For optical property examination, UV-vis absorption spectra were acquired with a UV-vis spectrophotometer (LAMBDA XLS, Perkin-Elmer). 3 mL of reactant solution was added to a 1 cm quartz cube. X-ray photoelectron spectroscopy (XPS, PHI 5600, US) was conducted to investigate the catalyst's chemical state, and the obtained spectra were calibrated by setting the C 1s peak at 284.8 eV. The crystalline structure of the samples was analyzed by X-ray diffraction (XRD; X’pert Philips MPD with a Panalytical X’celerator detector) using graphite monochromatized Cu Kα radiation of wavelength 1.5406 Å. Steady-state photoluminescence (PL) spectra were acquired on an FLS980 fluorescence spectrometer (Edinburgh Instruments) equipped with a 450 W xenon arc lamp as the excitation source. Time-resolved PL spectra were collected by the time-correlated single-photon counting (TCSPC) technique using the same spectrophotometer equipped with an EPL-375 picosecond pulsed diode laser (λem = 372 nm) with pulse width 66.5 ps and pulse repetition rate of 10 MHz with an average power of 75 μW in the nanosecond range or with a μF920H Xenon flash lamp in the microsecond range. PL decay curves were fitted by a stretched exponential function: I(t) = I_0_e^–^(t/τ)^β^, where τ and β are the PL lifetime and stretch parameters, respectively.


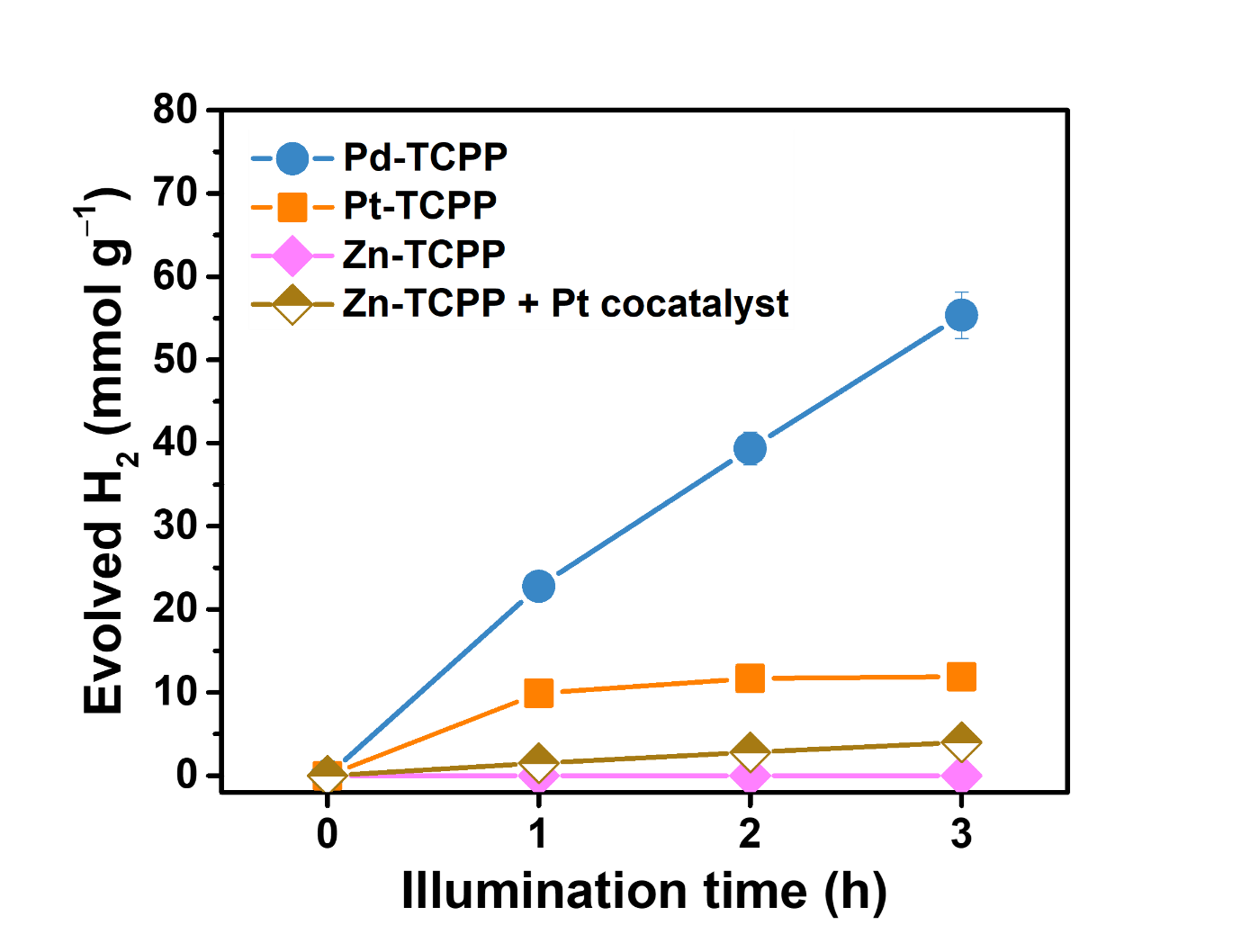


**Figure S1.** Photocatalytic H_2_ evolution (450 nm LED, 75 mW cm^−2^) of Zn–TCPP stacks with Pt cocatalyst (3 wt% H_2_PtCl_6_).

**
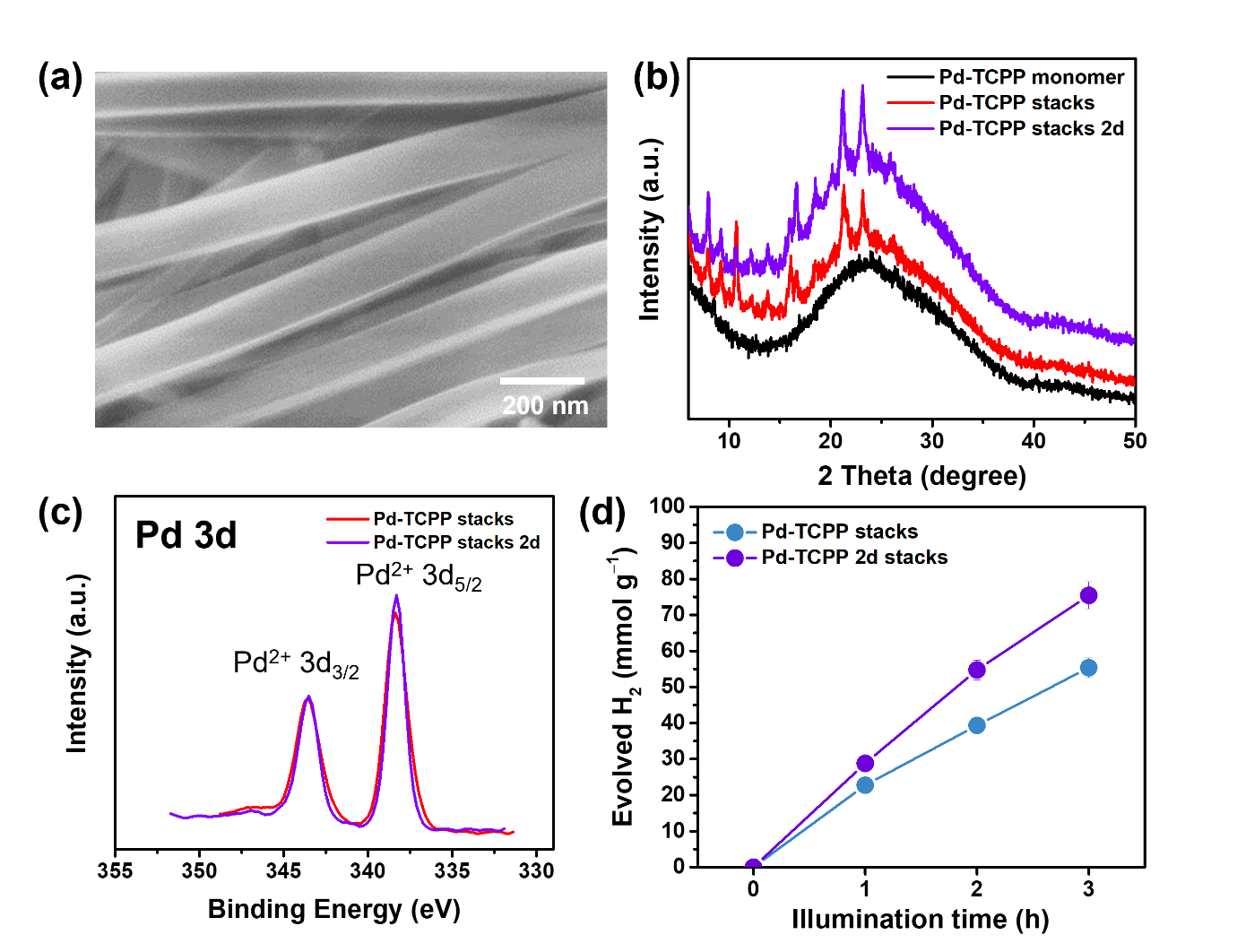
**

**Figure S2.** (a) SEM image, (b) XRD patterns, (c) XPS Pd 3d spectra, and (d) photocatalytic H_2_ evolution (450 nm LED, 75 mW cm^−2^) of Pd–TCPP stacks kept for 2 days (Pd–TCPP stacks 2d.)


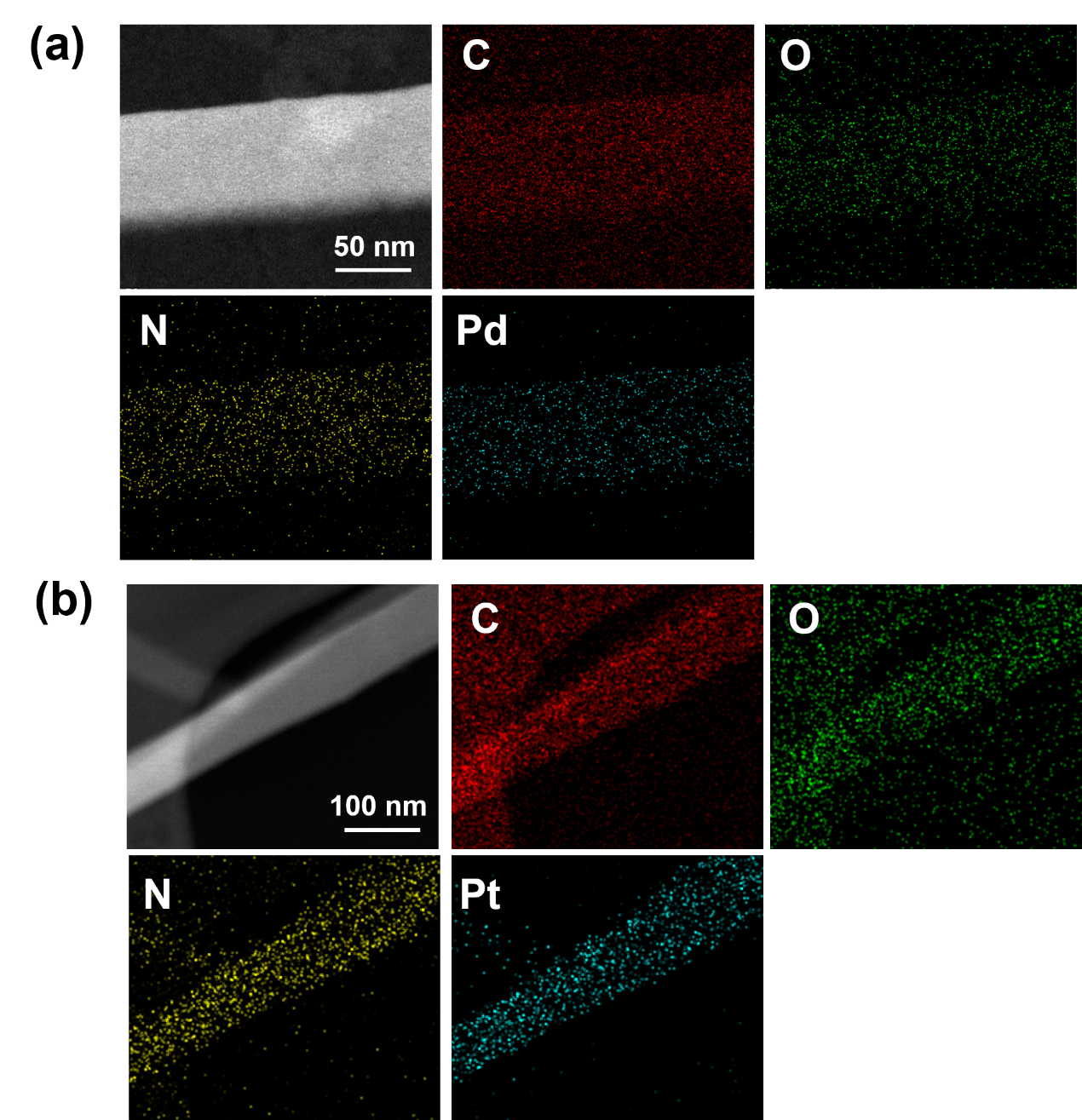


**Figure S3.** STEM–EDX mapping images of (a) Pd–TCPP stacks and (b) Pt–TCPP stacks.


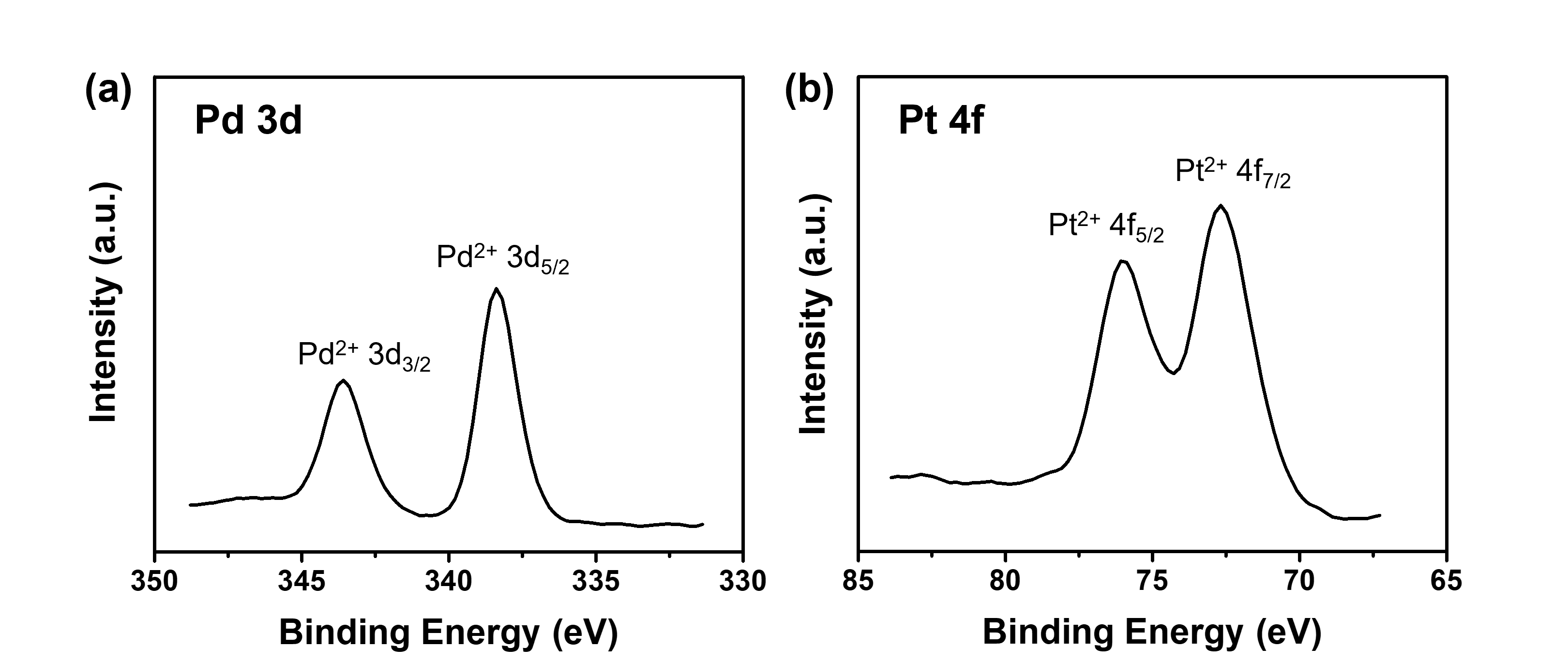


**Figure S4.** XPS: (a) Pd 3d spectra of Pd–TCPP stacks and (b) Pt 4f spectra of Pt–TCPP stacks.


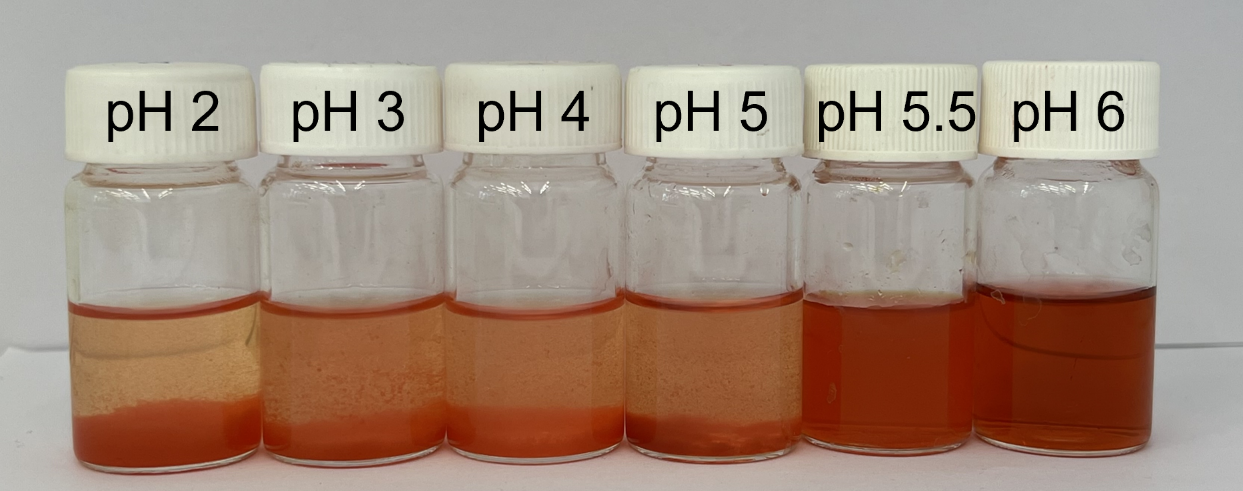


**Figure S5.** Pd–TCPP self-assembled stacks prepared at different pH values of MeOH/H_2_O aqueous solutions.


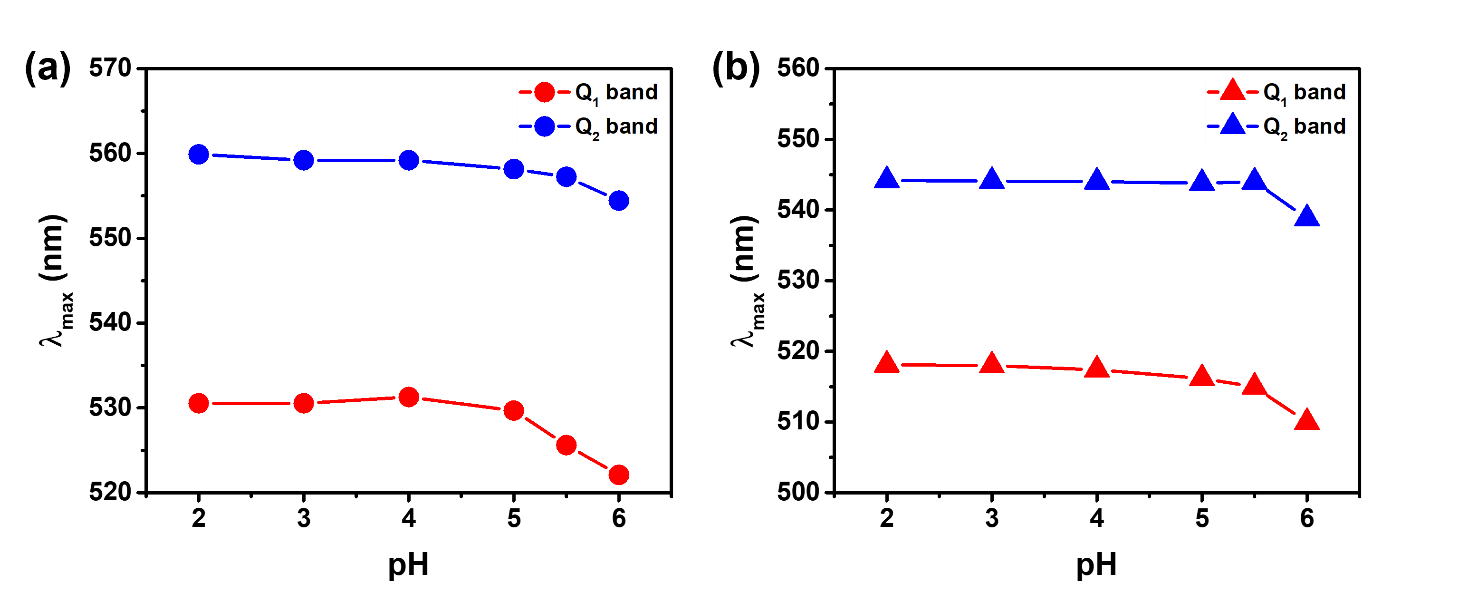


**Figure S6.** λ_max_ of Q-bands of (a) Pd–TCPP and (b) Pt–TCPP stacks at different pH values


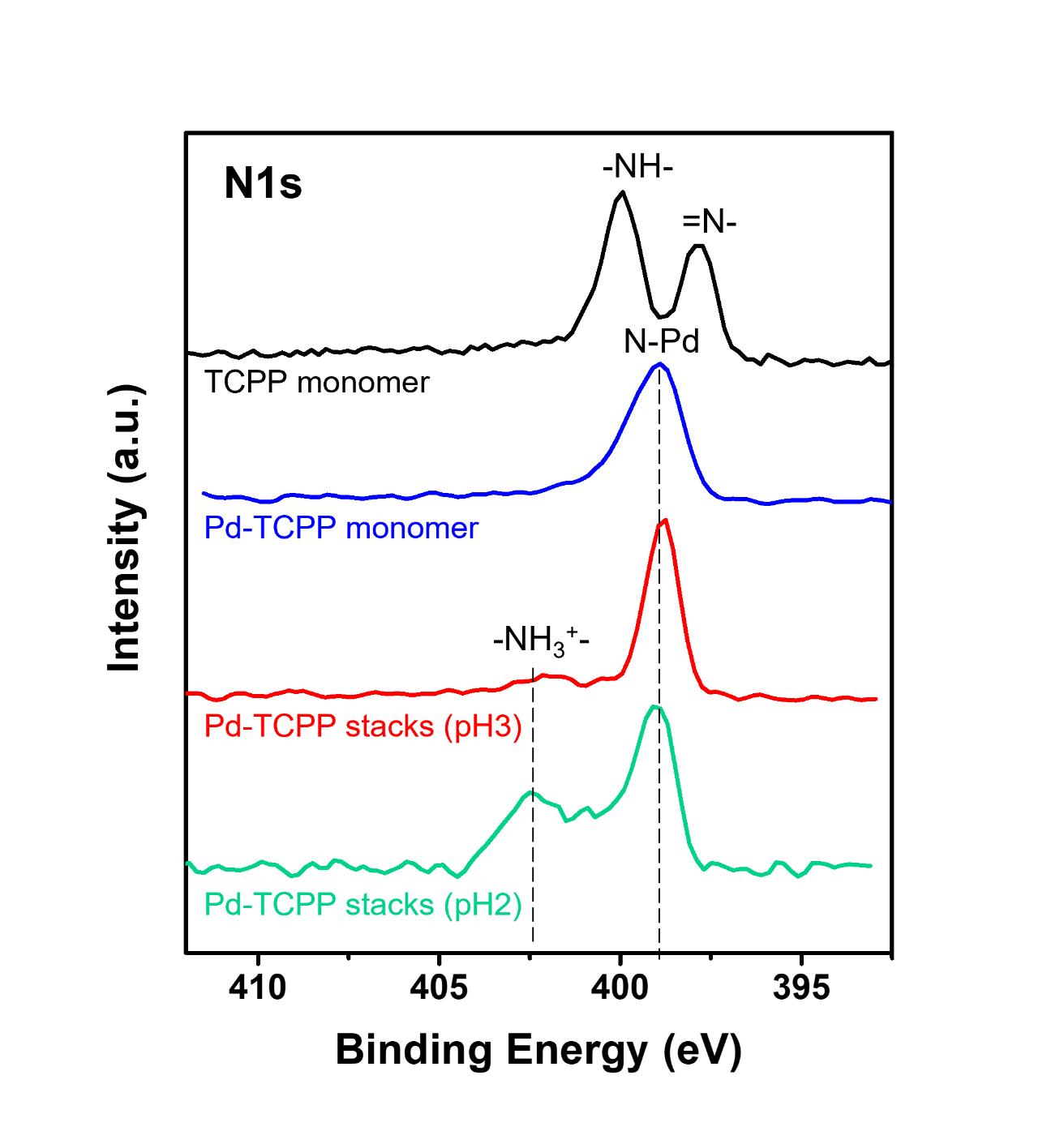


**Figure S7.** XPS N 1s spectra of TCPP monomer, Pd–TCPP monomer, and Pd–TCPP stacks (pH 2 and 3).


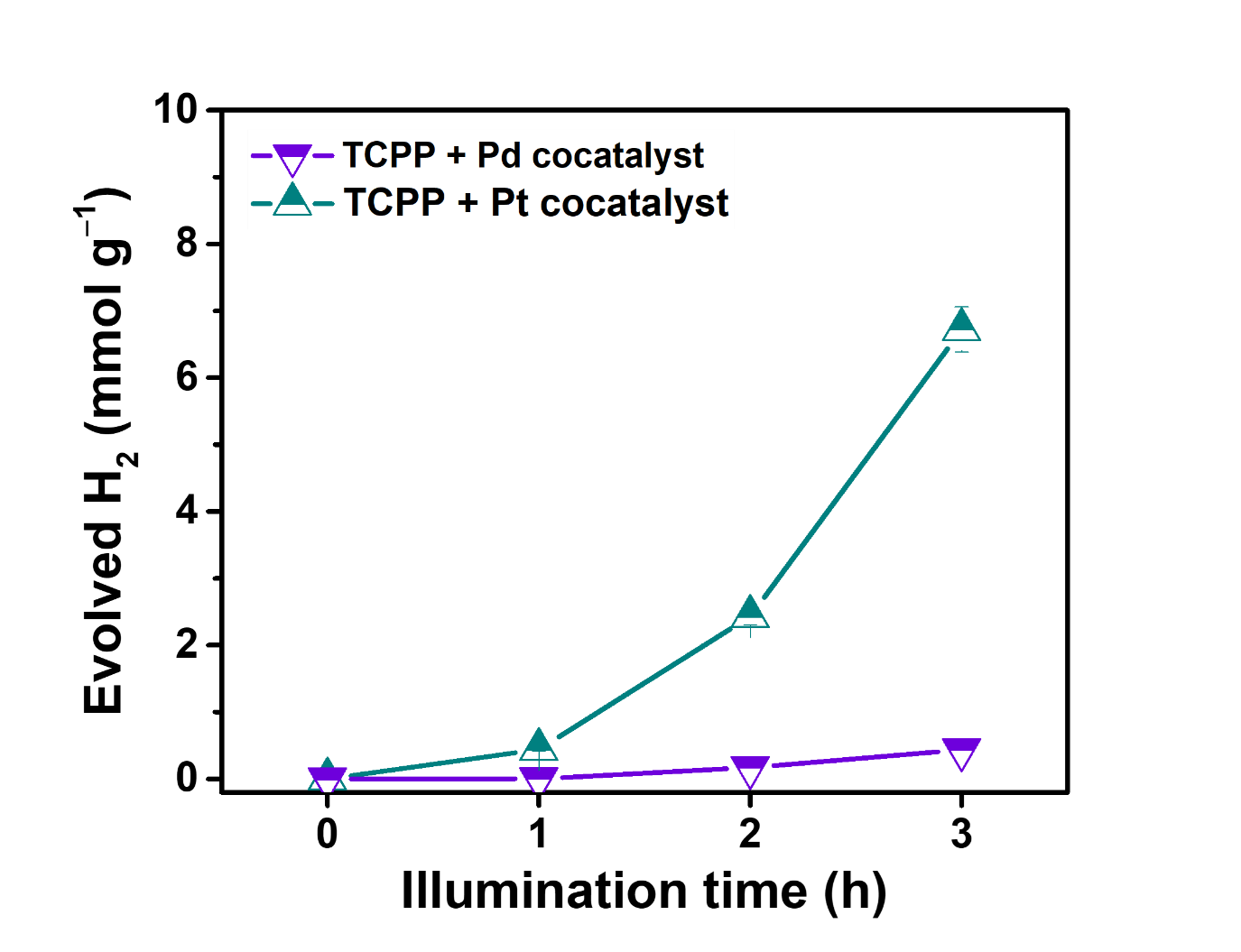


**Figure S8.** Photocatalytic H_2_ evolution (450 nm LED, 75 mW cm^−2^) of TCPP stacks with Pt and Pd co-catalyst (3 wt% H_2_PtCl_6_).


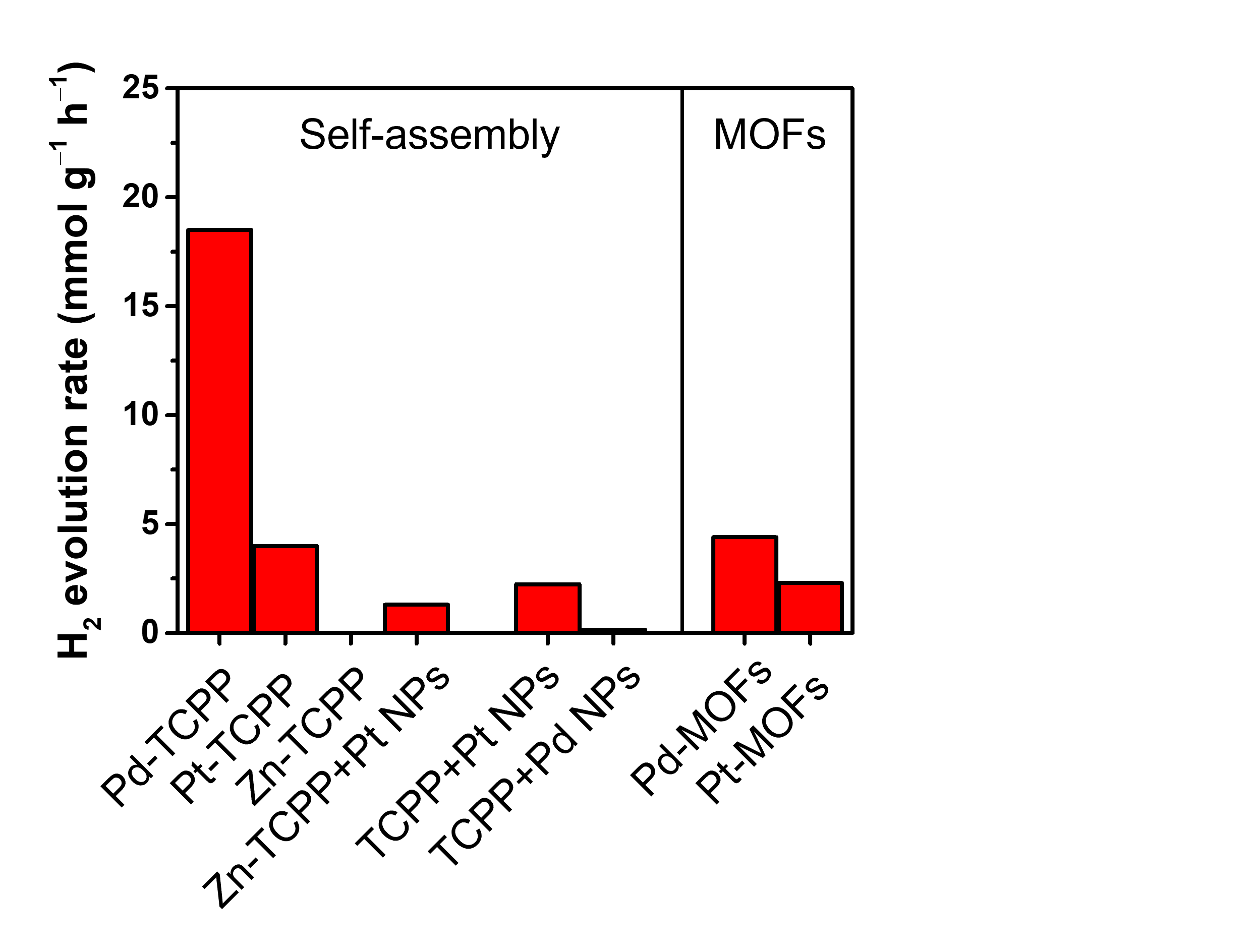


**Figure S9.** Photocatalytic H_2_ evolution rate (450 nm LED, 75 mW cm^−2^) of porphyrin-based self-assemblies and MOFs.


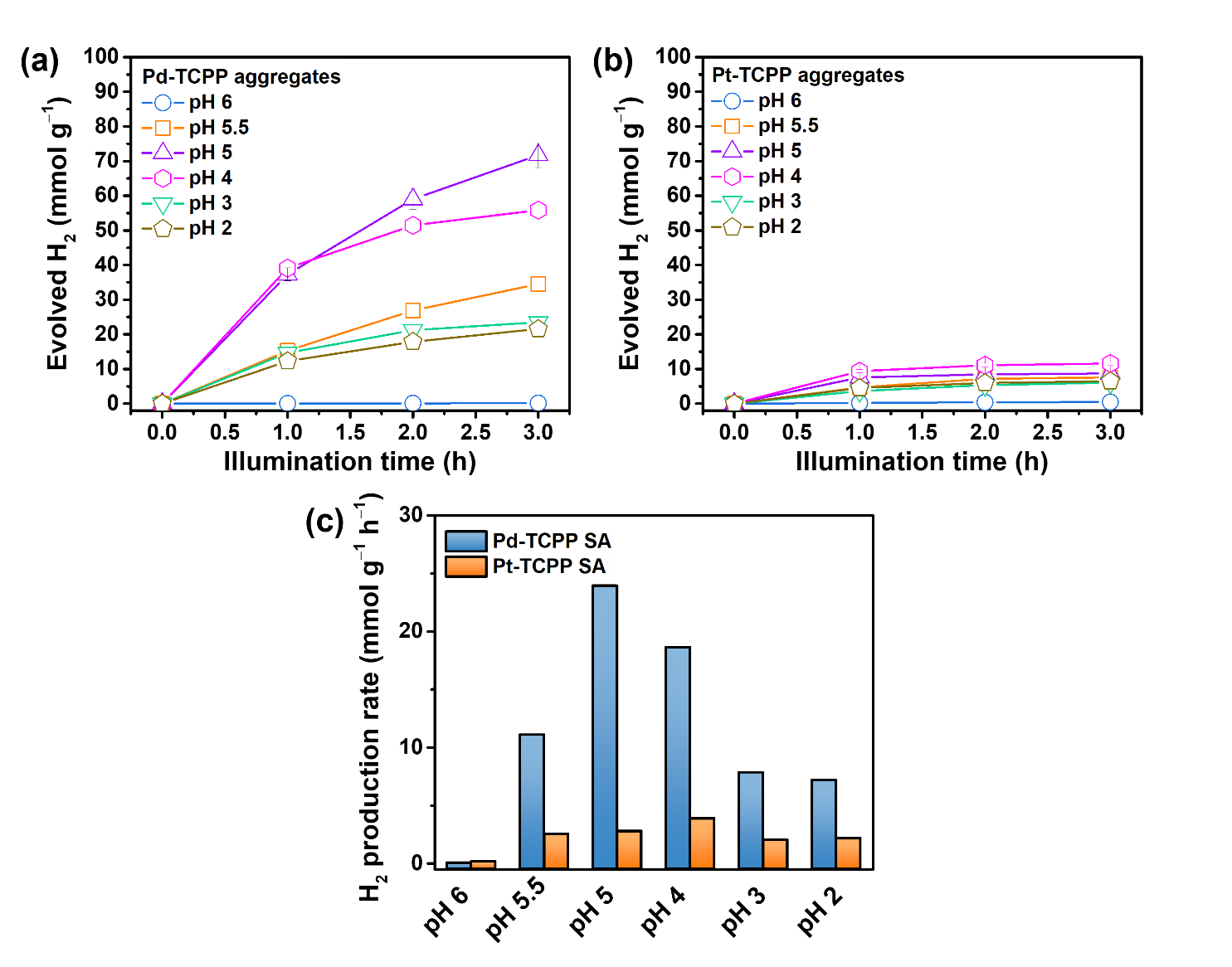


**Figure S10.** Photocatalytic H_2_ evolution (520 nm laser, 1.2 W cm^−2^) of (a) Pd–TCPP stacks and (b) Pt–TCPP stacks prepared in different pH solutions. (c) Corresponding photocatalytic H_2_ production rate.


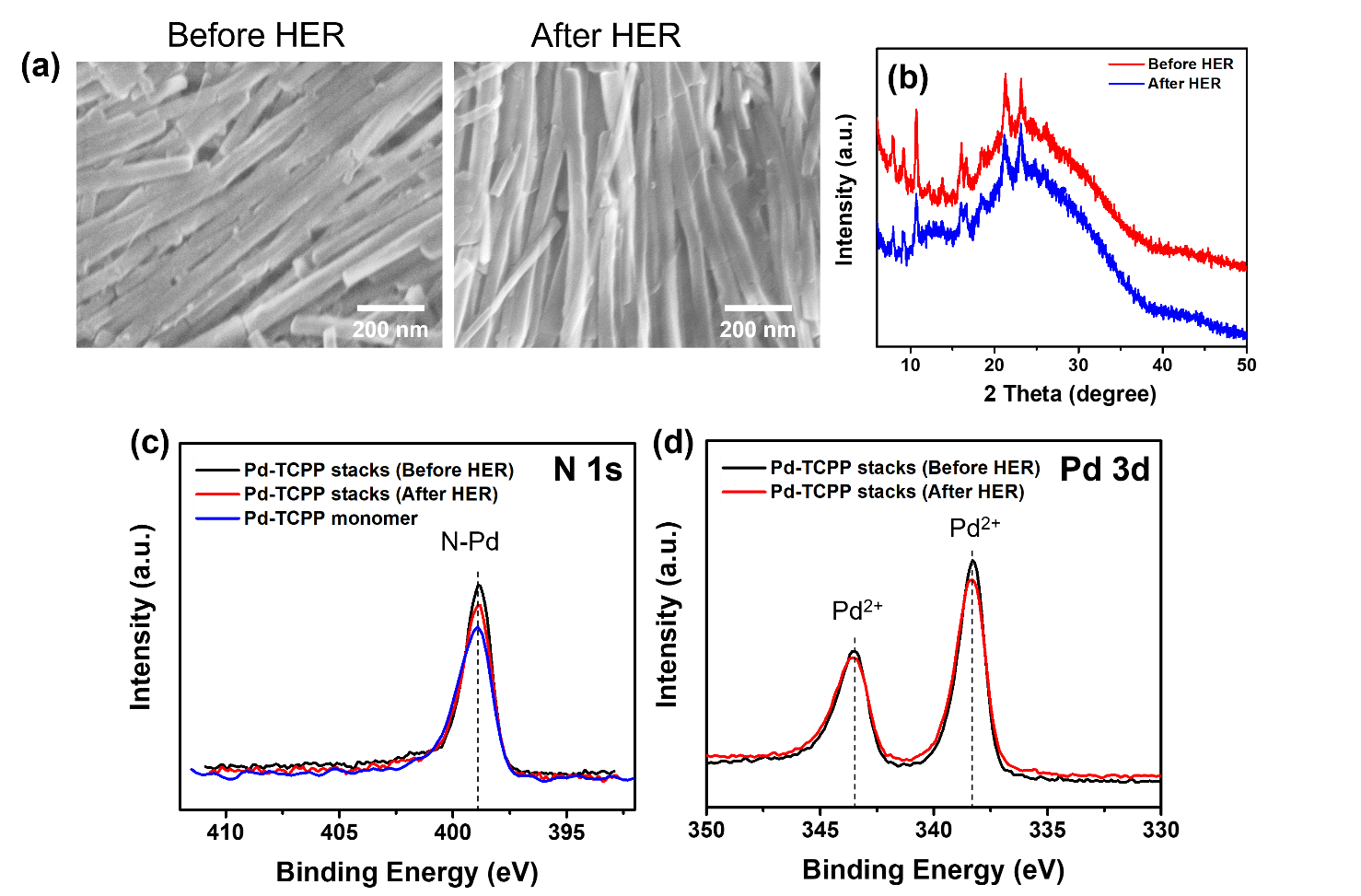


**Figure S11.** (a) SEM images, (b) XRD patterns, XPS (c) N 1s and (d) Pd 3d spectra of Pd–TCPP stacks (pH 4) after photocatalytic H_2_ evolution reaction.


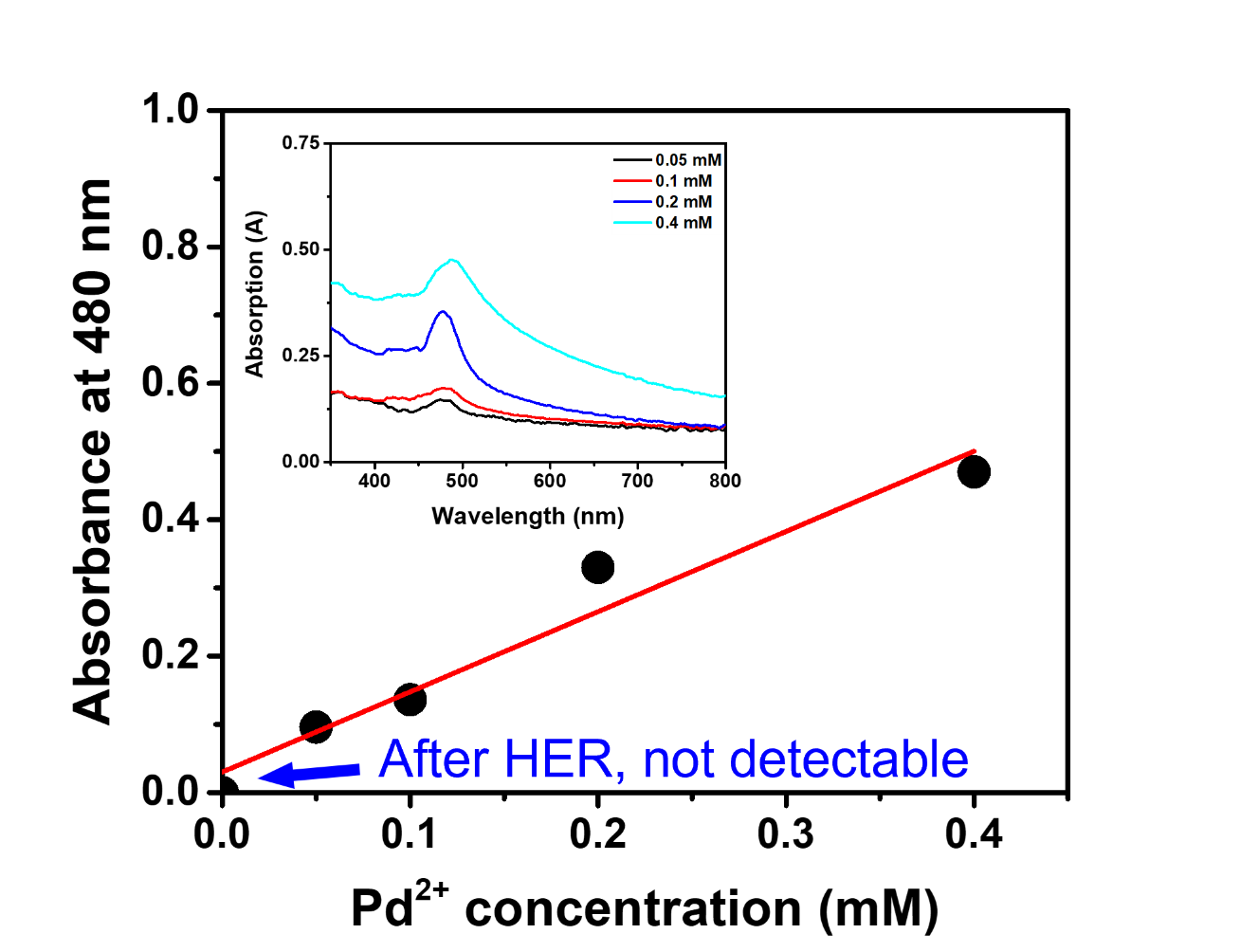


**Figure S12.** Pd^2+^ detection from Pd–TCPP stacks after photocatalytic H_2_ evolution reaction, using dimethylglyoxide (DMG) reagent (inset shows absorption spectra of Pd^2+^-DMG complex using different concentrations of Na_2_PdCl_4_)


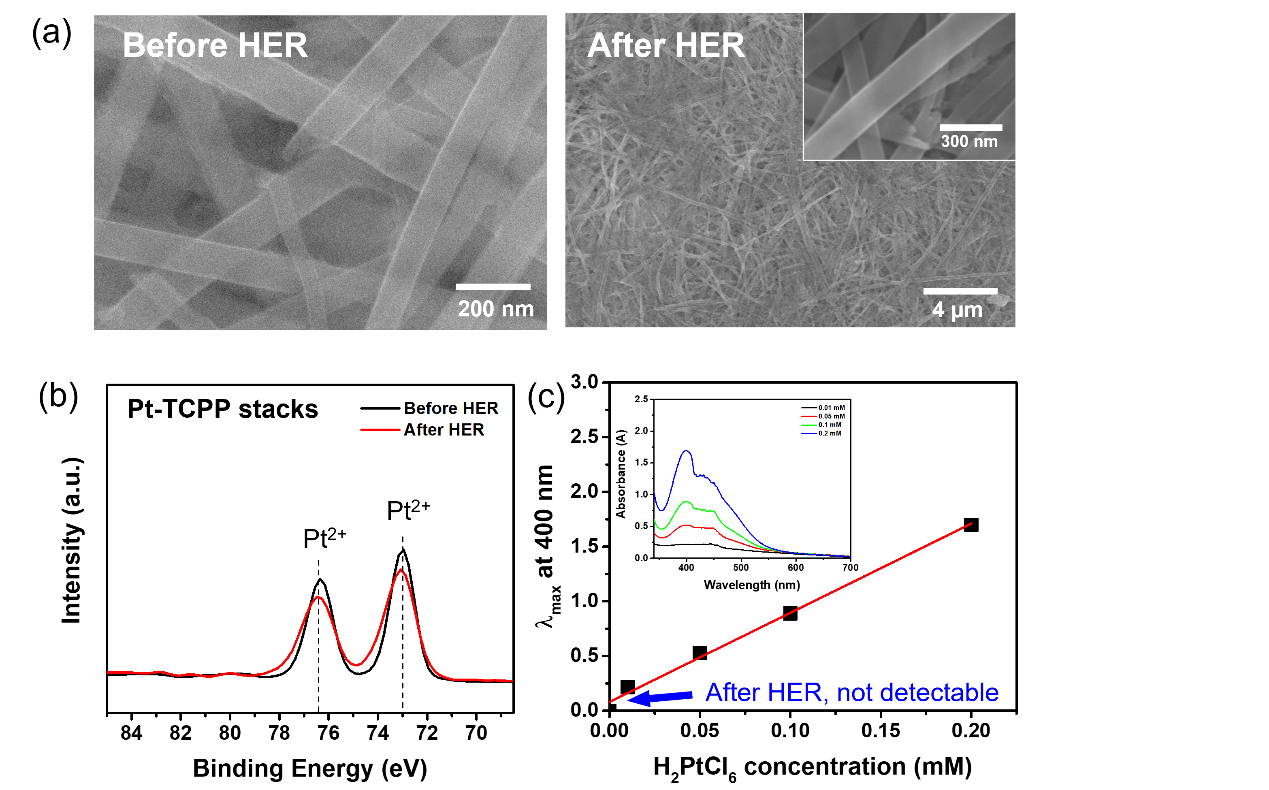


**Figure S13.** (a) SEM images and (b) XPS Pt 4f spectra of Pt–TCPP stacks before and after photocatalytic H_2_ evolution. (c) Pt^2+^ ion detection from Pt-TCPP stacks after photocatalytic H_2_ evolution using SnCl_2_ method. (inset shows absorption spectra of SnCl_2_-treated H_2_PtCl_6_).


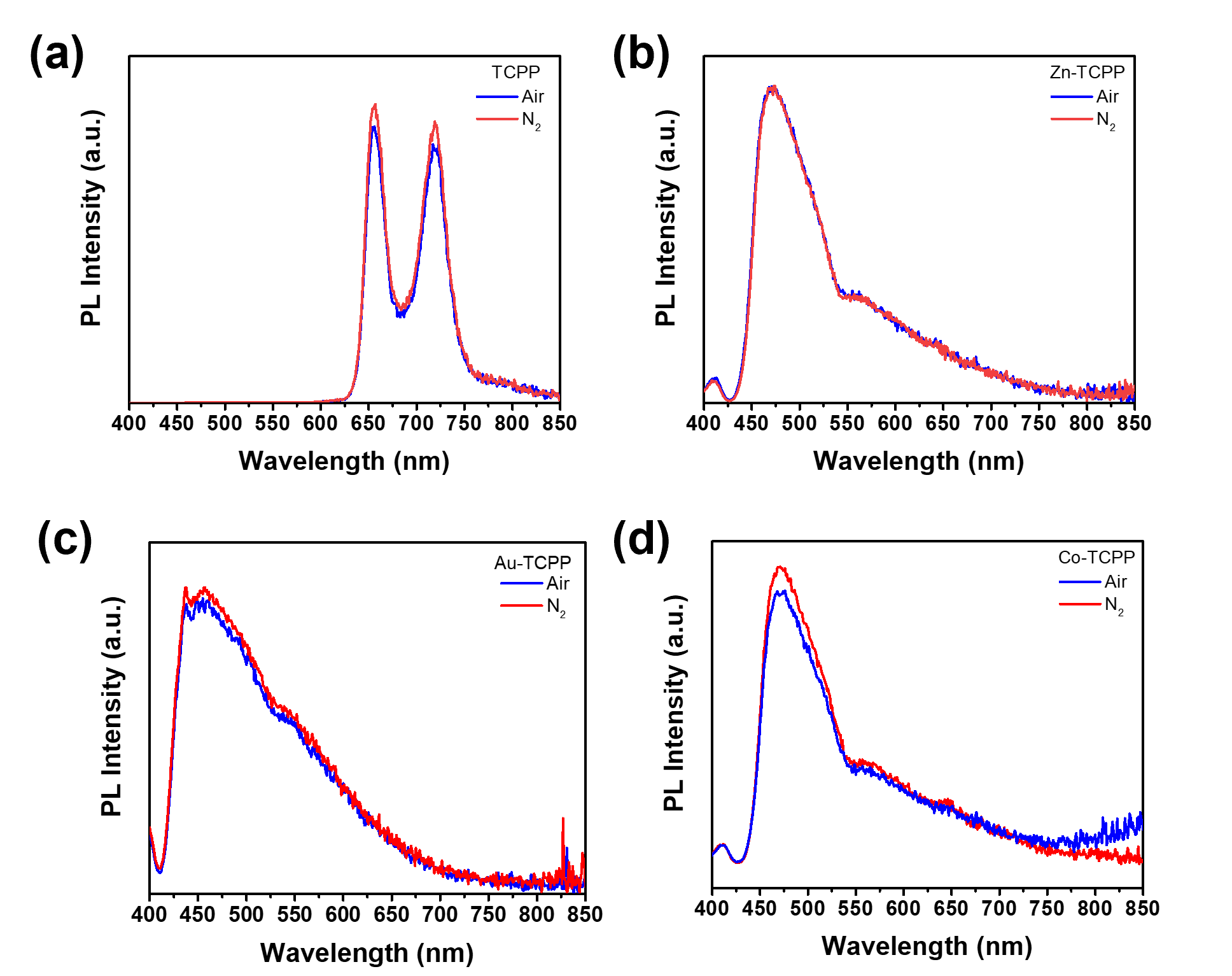


**Figure S14.** PL emission spectra of (a) metal-free TCPP, (b) Zn–TCPP, (c) Au–TCPP, and (d) Co–TCPP stacks under air and N_2_ atmosphere.


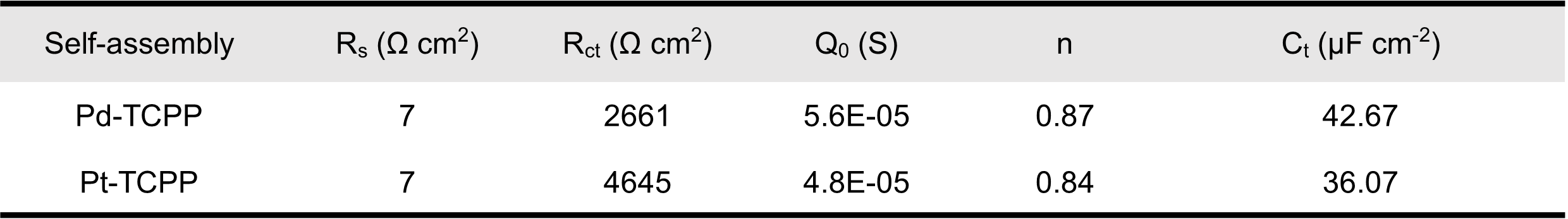


**Table S1.** EIS data of Pd–TCPP and Pt–TCPP stacks from Fig. 4d recorded in the frequency range of 10^–1^-10^6^ Hz in 0.1 M Na_2_SO_4_.


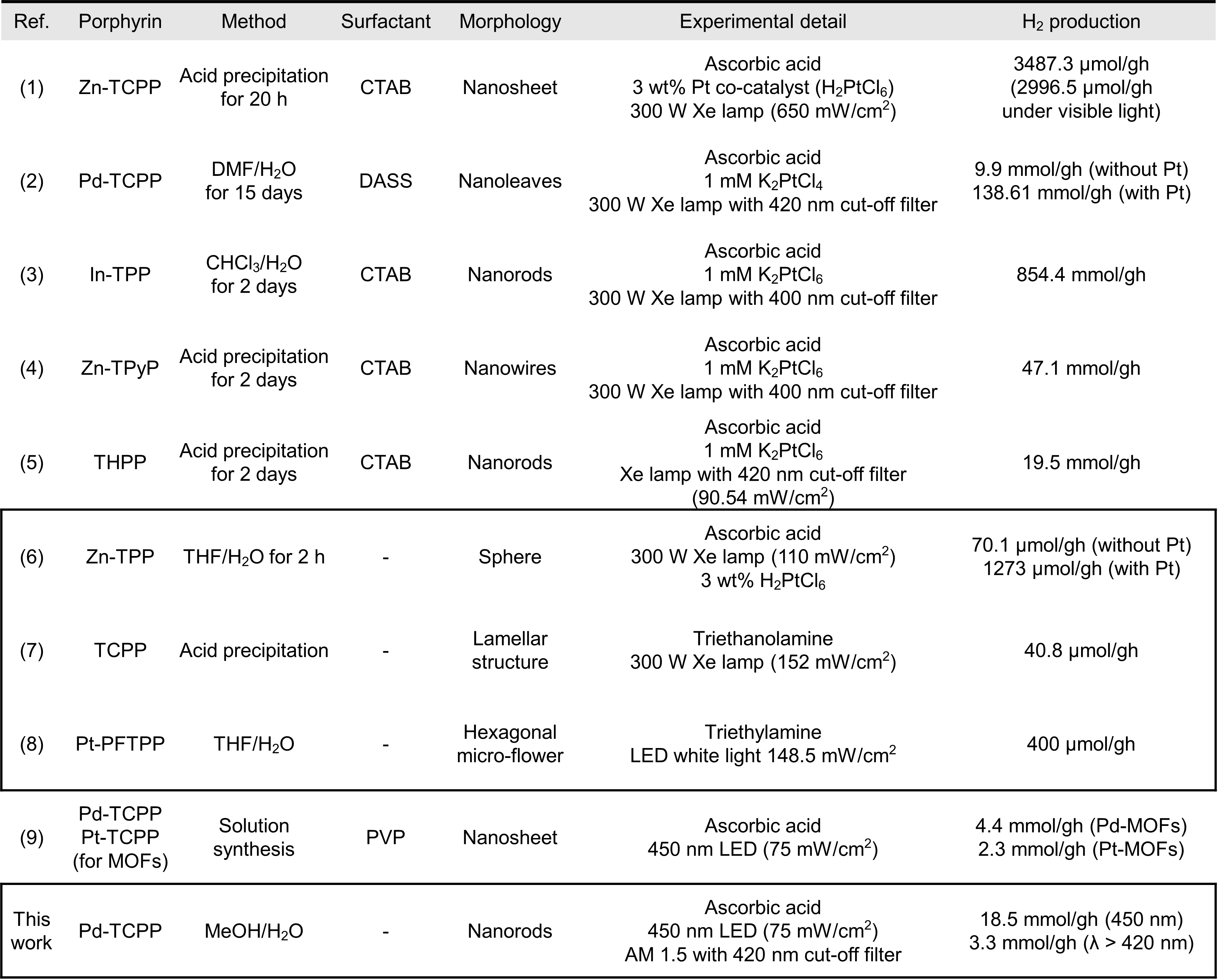


**Table S2.** Comparison of photocatalytic H_2_ production rate of porphyrin-based self-assemblies^1-8^ and MOFs^9^ (Solid box indicates the studies of porphyrin self-assemblies prepared without the use of surfactant).

**References**

(1) Jing, J.; Yang, J.; Zhang, Z.; Zhu, Y. Supramolecular zinc porphyrin photocatalyst with strong reduction ability and robust built‐in electric field for highly efficient hydrogen production. *Advanced Energy Materials* **2021**, *11* (29), 2101392.

(2) Cao, R.; Wang, G.; Ren, X.; Duan, P.-C.; Wang, L.; Li, Y.; Chen, X.; Zhu, R.; Jia, Y.; Bai, F. Self-assembled porphyrin nanoleaves with unique crossed transportation of photogenerated carriers to enhance photocatalytic hydrogen production. *Nano Letters* **2022**, *22* (1), 157-163.

(3) Liu, Y.; Wang, L.; Feng, H.; Ren, X.; Ji, J.; Bai, F.; Fan, H. Microemulsion-assisted self-assembly and synthesis of size-controlled porphyrin nanocrystals with enhanced photocatalytic hydrogen evolution. *Nano Letters* **2019**, *19* (4), 2614-2619.

(4) Wang, J.; Zhong, Y.; Wang, L.; Zhang, N.; Cao, R.; Bian, K.; Alarid, L.; Haddad, R. E.; Bai, F.; Fan, H. Morphology-controlled synthesis and metalation of porphyrin nanoparticles with enhanced photocatalytic performance. *Nano Letters* **2016**, *16* (10), 6523-6528.

(5) Zhang, N.; Wang, L.; Wang, H.; Cao, R.; Wang, J.; Bai, F.; Fan, H. Self-assembled one-dimensional porphyrin nanostructures with enhanced photocatalytic hydrogen generation. *Nano Letters* **2018**, *18* (1), 560-566.

(6) Sury, A.; Samuthirapandi, K.; Ghosh, S.; Kar, S.; Sarkar, S.; Kommula, B.; Bhattacharyya, S. Controlled Self‐Assembly of Zn‐Tetraphenylporphyrins for Efficient Photocatalytic Solar H_2_ Production and Simultaneous Organic Transformation to Valuable Chemicals. *ChemPhotoChem* **2024**, *8* (10), e202400105.

(7) Zhang, Z.; Zhu, Y.; Chen, X.; Zhang, H.; Wang, J. A full‐spectrum metal‐free porphyrin supramolecular photocatalyst for dual functions of highly efficient hydrogen and oxygen evolution. *Advanced Materials* **2019**, *31* (7), 1806626.

(8) Bodedla, G. B.; Piradi, V.; Thor, W.; Wong, K.-L.; Zhu, X.; Wong, W.-Y. Self-assembly of Pt (II)-tetrakis (pentafluorophenyl) porphyrin via F⋯ F interaction for efficient cocatalyst-free photocatalytic hydrogen evolution. *Journal of Materials Chemistry A* **2024**, *12* (5), 2924-2931.

(9) Kim, J. H.; Wu, S.; Zdrazil, L.; Denisov, N.; Schmuki, P. 2D Metal–Organic Framework Nanosheets based on Pd‐TCPP as Photocatalysts for Highly Improved Hydrogen Evolution. *Angewandte Chemie International Edition* **2024**, *63* (7), e202319255.
